# Input-specific modulation of murine nucleus accumbens differentially regulates hedonic feeding

**DOI:** 10.1038/s41467-021-22430-7

**Published:** 2021-04-09

**Authors:** Daniel J. Christoffel, Jessica J. Walsh, Boris D. Heifets, Paul Hoerbelt, Sophie Neuner, Gordon Sun, Vinod K. Ravikumar, Hemmings Wu, Casey H. Halpern, Robert C. Malenka

**Affiliations:** 1 Nancy Pritzker Laboratory, Department of Psychiatry and Behavioral Sciences, Stanford University, Stanford, CA USA; 2 Department of Anesthesiology, Perioperative and Pain Medicine, Stanford University School of Medicine, Stanford, CA USA; 3 Department of Neurosurgery, Stanford University School of Medicine, Stanford, CA USA

**Keywords:** Obesity, Reward

## Abstract

Hedonic feeding is driven by the “pleasure” derived from consuming palatable food and occurs in the absence of metabolic need. It plays a critical role in the excessive feeding that underlies obesity. Compared to other pathological motivated behaviors, little is known about the neural circuit mechanisms mediating excessive hedonic feeding. Here, we show that modulation of prefrontal cortex (PFC) and anterior paraventricular thalamus (aPVT) excitatory inputs to the nucleus accumbens (NAc), a key node of reward circuitry, has opposing effects on high fat intake in mice. Prolonged high fat intake leads to input- and cell type-specific changes in synaptic strength. Modifying synaptic strength via plasticity protocols, either in an input-specific optogenetic or non-specific electrical manner, causes sustained changes in high fat intake. These results demonstrate that input-specific NAc circuit adaptations occur with repeated exposure to a potent natural reward and suggest that neuromodulatory interventions may be therapeutically useful for individuals with pathologic hedonic feeding.

## Abstract (web summary)

Hedonic feeding occurs in the absence of metabolic need and plays a critical role in the excessive feeding that underlies obesity. The authors show that optogenetic manipulation of NAc inputs from the prefrontal cortex versus inputs from the anterior paraventricular nucleus of the thalamus has opposite effects on high fat intake.

## Introduction

Over the last three decades, the rate of obesity and comorbid eating disorders has increased substantially^1^. While the evolutionarily conserved impulse of gorging on fatty foods was once advantageous, overconsumption of palatable foods is a primary driver of the current obesity epidemic. One reason individuals struggle to abstain from overeating is that the value of palatable food remains high in the absence of physiological need, leading to hedonic feeding^2^. The cycles of abstinence and relapse that occur during hedonic binge-eating behaviors, often driven by pathological cravings, provide suggestive evidence that the neural circuits mediating pathological hedonic feeding may overlap with those underlying drug addiction^2–4^. However, while the synaptic and circuit adaptations caused by administration of drugs of abuse have been the object of intense study for several decades^5,6^, the extent to which similar changes occur during pathological feeding remains unclear.

The nucleus accumbens (NAc), a major hub of the brain’s reward circuitry, is well established to play a critical role in feeding behavior^4,7^. Indeed, human brain imaging studies suggest that NAc reactivity to a food cue predicts future weight gain^8^, while obese individuals exhibit dysfunction of the striatum and frontal cortex similar to that observed in chronic cocaine users^4^. Here we begin to examine how specific excitatory inputs to the NAc influence a mouse model of binge-eating behavior^9^. We find that optogenetic manipulation of NAc inputs from the prefrontal cortex (PFC) versus inputs from the anterior paraventricular nucleus of the thalamus (aPVT) has opposite effects on high fat intake. Furthermore, the adaptations at synapses made by these inputs onto NAc medium spiny neuron (MSN) subtypes due to prolonged high fat intake are distinct and modifying synaptic strength via plasticity inducing protocols causes sustained alterations in high fat intake.

## Results

### Bidirectional modulation of high fat intake by discrete NAc inputs

To assess NAc input-specific regulation of palatable food consumption, we employed a limited-access high fat exposure model, where sated mice develop stable levels of intake during the first week of exposure. Mice consume ~25% of their total daily caloric intake in this 1-h period during the light cycle, a behavior that is used to model binge-eating behavior^9^. While by days 10–12 of high fat exposure all mice reach stable intake, there is significant individual variability in the magnitude of initial and final high fat intake (*n* = 112, Supplementary Fig. 1a). Individuals in the lower two quartiles of initial consumption show the largest increase in the amount of high fat consumed, while individuals in the highest quartile of initial consumption show a much more modest increase (Supplementary Fig. 1b).

**Fig. 1:**
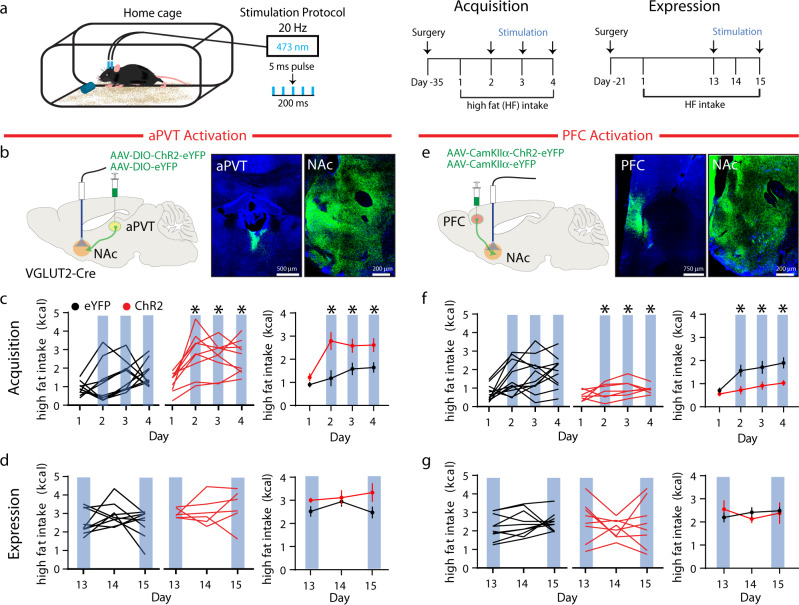
Activation of distinct NAc glutamatergic inputs has opposing effects on high fat intake. **a** Schematic of experimental setup (left); experimental timeline (right). **b** Schematic of viral injection and ferrule implant for aPVT input stimulation (left); representative images of ChR2 infection in the aPVT and NAc (right). **c**, **d** Quantification of high fat intake during acquisition (**c**: *F*_1,17_ = 13.1 *P* = 0.002, *n* = 10, 9 mice/group) and expression period (**d**: *F*_1,13_ = 4.03, *P* = 0.07, *n* = 9, 6 mice/group) in eYFP control (black) and ChR2 expressing (red) mice. In this and all subsequent figure panels, blue bars signify blue light stimulation; left panels illustrate individual subjects; far right panel displays mean ± s.e.m. **e** Schematic of viral injection and ferrule implant for PFC input stimulation (left); representative images of ChR2 infection in the PFC and NAc (right). **f**, **g** Quantification of high fat intake during acquisition (**f**: *F*_1,18_ = 9.03, *P* = 0.008, *n* = 10, 8 mice/group) and expression period (**g**: *F*_1,16_ = 0.001, *P* > 0.05, *n* = 10, 8 mice/group). Data are mean ± s.e.m. *P < 0.05, two-way ANOVA with Sidak’s multiple comparison post hoc test. The schematic of the mouse brain in this and all subsequent figures has been adapted with permission from Paxinos & Franklin^57^. Source data are provided as a Source Data file.

Several excitatory inputs to the NAc are involved in feeding behavior^10–13^. We chose to investigate the role of NAc inputs from the aPVT and PFC because they both appear to regulate the consumption of drugs of abuse and palatable food yet have distinct reinforcing properties when acutely activated^14–19^. To optogenetically activate excitatory, glutamatergic inputs in the NAc from the aPVT, we injected a Cre-dependent channelrhodopsin (ChR2), AAV-DIO-ChR2-eYFP or AAV-DIO-eYFP (as a control) into the aPVT of VGLUT2-Cre mice and delivered 474 nm blue light at 20 Hz bilaterally through optical fibers implanted to preferentially target the shell region of the NAc throughout the entire exposure period (Fig. 1a, b and Supplementary Fig. 2a). We assessed the effects of aPVT input activation on two distinct phases of high fat intake: an acquisition period, defined as days 1–4 of exposure, during which mice ramp up to stable levels of intake, and an expression period, defined as days 13–15, during which mice readily consume the high-fat pellet and intake levels are relatively stable (Fig. 1a). Activation of aPVT→NAc inputs during the acquisition period on days 2–4 rapidly increased intake to levels seen during stable, chronic intake (Fig. 1c). In contrast, activation of these inputs on days 13 and 15, during the expression period, did not significantly increase high fat intake when compared to day 14 when no stimulation was applied (Fig. 1d).

To activate glutamatergic PFC inputs in the NAc, we injected AAV-CamKIIα-ChR2-eYFP or AAV-CamKIIα-eYFP into the PFC of wild-type C57Bl/6J mice and again provided 20 Hz blue light to the NAc bilaterally during acquisition and expression periods (Fig. 1a, e and Supplementary Fig. 2b). In contrast to the effects of activating aPVT → NAc inputs, activation of PFC → NAc inputs reduced high fat intake during the acquisition period on days 2–4 (Fig. 1f). However, activation of these inputs had no effect on high fat intake after it had stabilized during the expression period on days 13 and 15 compared to day 14 (Fig. 1g). To verify that optogenetic activation of inputs was limited to altering NAc activity and not adjacent regions, we stimulated the inputs within the NAc ~1 h prior to perfusion and assessed cFos levels. Activation of either aPVT or PFC inputs increased the number of cells expressing cFos in the NAc, primarily the shell region, but not in the immediately adjacent dorsal striatum (Supplementary Fig. 3). Together these data suggest that the aPVT inputs to NAc can promote, whereas the PFC inputs can suppress, the acquisition of binge-eating behavior.

**Fig. 2:**
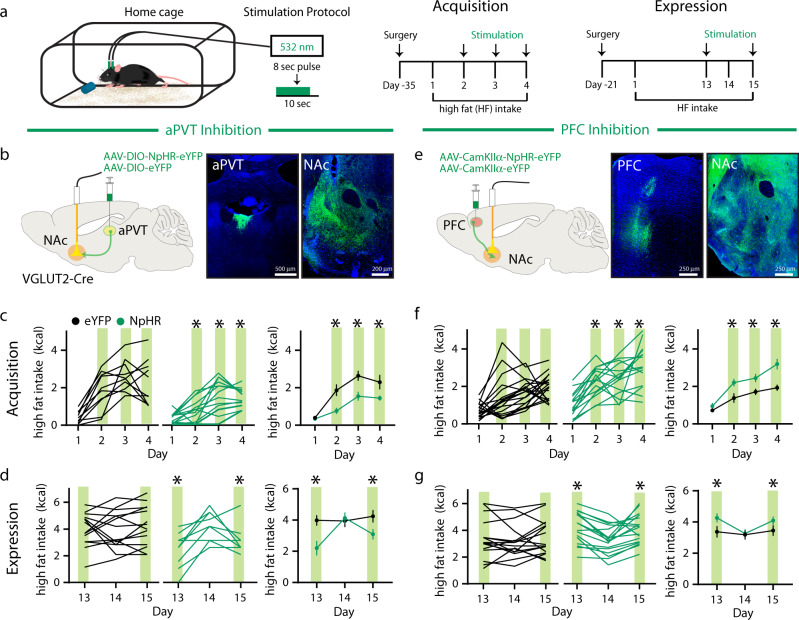
Inhibition of distinct NAc glutamatergic inputs has opposing effects on high fat intake. **a** Schematic of experimental setup (left); experimental timeline (right). **b** Schematic of viral injection and ferrule implant for aPVT input inhibition (left); representative images of NpHR infection in the aPVT and NAc (right). **c**, **d** Quantification of high fat intake during acquisition (**c**: *F*_3,66_ = 3.71, *P* = 0.01, *n* = 10,14 mice/group) and expression period (**d**: *F*_2,44_ = 8.04, *P* = 0.001, *n* = 14,10 mice/group) in eYFP control (black) and NpHR expressing (green) mice. Green bars signify green light stimulation. **e** Schematic of viral injection and ferrule implant for PFC input inhibition (left); representative images of NpHR infection in the PFC and NAc (right). **f**, **g** Quantification of high fat intake during acquisition (**f**: *F*_3,99_ = 3.23, *P* = 0.03, *n* = 19,16 mice/group) and expression period (**g**: *F*_2,62_ = 2.95, *P* = 0.047, *n* = 16,17 mice/group). Data are ±s.e.m. *P < 0.05, two-way ANOVA with Sidak’s multiple comparison post hoc test. Source data are provided as a Source Data file.

To assess the necessity of aPVT and PFC inputs activity in the acquisition and expression of high fat intake, we used an identical experimental strategy but expressed the inhibitory opsin, halorhodopsin (NpHR), while delivering 532 nm green light bilaterally to the NAc with an 80% duty cycle throughout the entire exposure period (Fig. 2a and Supplementary Fig. 4). Inhibition of aPVT→NAc inputs reduced high fat intake during both the acquisition (days 2–4) and expression (days 13 and 15) periods (Fig. 2b–d). Inhibition of PFC → NAc inputs had the opposite effects, increasing high fat intake during both periods (Fig. 2–g). These results provide further evidence that aPVT → NAc and PFC → NAc input activity have opposite effects on high fat intake and that both exert bidirectional control over this motivated behavior.

These results beg the question, “How do two glutamatergic inputs differentially affect high fat intake?”. One possibility is that the two inputs innervate different NAc subregions. Indeed, PVT and basolateral amygdala inputs have been shown to have different effects on behavior and innervate distinct areas of the NAc^20^. To begin addressing this question, we expressed distinct fluorophores in the aPVT and PFC, then mapped their innervation pattern throughout the rostro-caudal extent of defined NAc subregions. aPVT and PFC inputs innervated distinct areas of the NAc shell, while having overlapping innervation of the core (Supplementary Fig. 5a–c). A pixelwise analysis of input colocalization revealed a negative correlation in the NAc shell, with preferential innervation of one input in distinct areas of the shell (Supplementary Fig. 5d–f).

### aPVT and PFC input activity do not influence standard chow intake

Previous work in rats demonstrated that blockade of AMPA receptors in the NAc shell robustly increases the intake of standard chow^21,22^. These findings raise the question of whether optogenetic manipulations of the aPVT and PFC inputs to the NAc influence intake of standard chow, which presumably does not have the large appetitive value of high fat pellets. In contrast to the effects on high fat intake, activation, and inhibition of either input had no effect on intake of a standard chow pellet, which was presented for 1 h at the same time of day instead of a high fat pellet (Supplementary Fig. 6). A limitation of these experiments is that the levels of standard chow consumed were low, perhaps making it difficult to detect effects of the optogenetic manipulations. Therefore, to further explore this topic we switched to a chemogenetic inhibition strategy, which allowed prolonged inhibition of input activity during the animals’ dark cycle when they consume most of their calories.

**Fig. 3:**
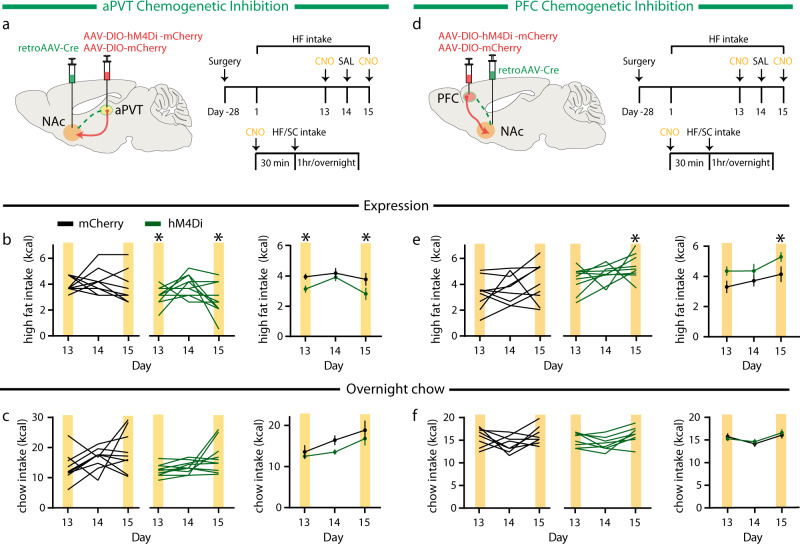
Inhibition of distinct NAc projecting neurons selectively regulates high fat intake. **a** Schematic of viral injection for NAc projecting aPVT neuron inhibition (left); experimental timeline (right). **b** Quantification of high fat intake during expression period (*F*_1,18_ = 4.94, *P* = 0.03, *n* = 10 mice/group) in mCherry control (black) and hM4Di expressing (green) mice. Yellow bars signify CNO administration. **c** Quantification of overnight standard chow intake during expression period (*F*_1,18_ = 3.5, *P* > 0.05, *n* = 10 mice/group) in mCherry control (black) and hM4Di expressing (green) mice. **d** Schematic of viral injection for NAc projecting PFC neuron inhibition (left); experimental timeline (right). **e** Quantification of high fat intake during expression period (*F*_1,17_ = 5.27, *P* = 0.03, *n* = 9, 10 mice/group) in mCherry control (black) and hM4Di expressing (green) mice. **f** Quantification of overnight standard chow intake during expression period (*F*_1,17_ = 0.19, *P* > 0.05, n = 9, 10 mice/group) in mCherry control (black) and hM4Di expressing (green) mice. Data are ±s.e.m. *P < 0.05, two-way ANOVA with Sidak’s or Tukey’s multiple comparison post hoc test. Source data are provided as a Source Data file.

To selectively inhibit NAc projecting aPVT neurons, we employed a two-virus intersectional strategy, injecting a retroAAV-Cre into the NAc and an AAV expressing a Cre-dependent inhibitory designer receptor exclusively activated by designer drugs (hM4Di) or an mCherry control into the aPVT. Previous studies report that the aPVT does not contain GABAergic neurons^23^ and there is no staining for either GAD gene in the Allen Brain Atlas. Nevertheless, to confirm the paucity of GABAergic neurons in the aPVT, we stained brain sections with an anti-GAD65 antibody (Ab), which clearly labeled neurons in the hippocampus while yielding no detectable labeling in the aPVT (Supplementary Fig. 7). Thus, it is very unlikely that our two-virus intersectional approach accessed NAc projecting PVT neurons that were GABAergic and not glutamatergic. To test the efficacy of this manipulation, we administered CNO (10 mg/kg) intraperitoneally (IP) 30 min prior to the 1-h high fat pellet exposure on days 13 and 15, with control saline on day 14 (Fig. 3a). Similar to optogenetic inhibition, this decreased high fat intake (Fig. 3b). We then administered CNO again 30 min prior to the dark cycle and observed no effect on overnight standard chow consumption (Fig. 3c). We used the same two-virus intersectional strategy to selectively inhibit NAc projecting PFC neurons (Fig. 3d). In agreement with our optogenetic findings, inhibition of PFC → NAc neurons caused a significant increase in high fat intake, albeit on the second, but not first, day of CNO administration (Fig. 3e). However, this chemogenetic inhibition had no effect on overnight chow intake (Fig. 3f). These results suggest that manipulation of the activity of these inputs preferentially regulates intake of a novel, highly palatable food, so-called hedonic feeding.

### aPVT and PFC inputs to NAc selectively regulate the rewarding properties of high fat

**Fig. 4:**
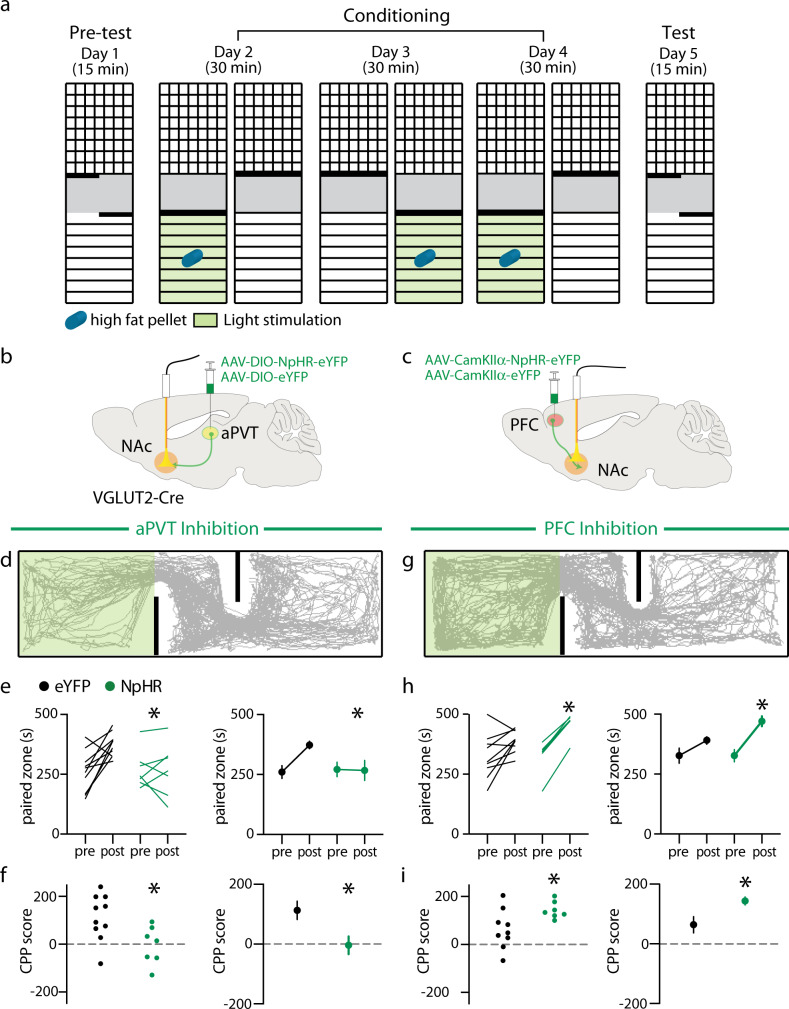
Inhibition of distinct NAc glutamatergic inputs regulates high fat place preference. **a** Schematic of experimental setup and design. **b** Schematic of viral injection and ferrule implant for aPVT input inhibition. **c** Schematic of viral injection and ferrule implant for PFC input inhibition. **d** Trajectory of a typical animal during test that received aPVT input inhibition during the conditioning phase. **e**, **f** Quantification of time spent in the paired chamber (**e**: *F*_1,15_ = 6.948, *P* = 0.02, *n* = 10,7 mice/group) and CPP score for paired chamber (**f**: *t* = 2.636, *P* = 0.02, *n* = 10,7 mice/group) in eYFP control (black) and NpHR expressing (green) mice. **g** Trajectory of a typical animal during test that received PFC input inhibition during the conditioning phase. **h**, **i** Quantification and time spent in paired chamber (**h**: *F*_1,14_ = 5.843, *P* = 0.03, *n* = 9, 7 mice/group) and CPP score for paired chamber (**i**: *t* = 2.417, *P* = 0.03, *n* = 9, 7 mice/group) in eYFP control (black) and NpHR expressing (green) mice. Data are ±s.e.m. *P < 0.05, **e**, **h** two-way ANOVA with Sidak’s multiple comparison post hoc test, **f**, **i** Student’s *t* test. Source data are provided as a Source Data file.

To assess how the activity of these inputs regulates high fat seeking behavior we performed a conditioned place preference (CPP) assay, a well-established protocol to assess the rewarding properties of a stimulus. For three consecutive days, animals were placed in a chamber containing a high fat pellet for 30 min during which aPVT → NAc inputs or PFC → NAc inputs were inhibited optogenetically (Fig. 4a–c). Similar to the effects of inhibition during high fat intake experiments, optogenetic inhibition of aPVT → NAc inputs blocked the preference for the high fat paired chamber, which was observed in the eYFP controls (Fig. 4d–f). In contrast, inhibition of PFC → NAc inputs enhanced the preference for the high fat paired chamber (Fig. 4g–i). To test if these findings might be influenced by the fact that inhibition of either input modified the valence of the paired zone, we repeated these experiments in the absence of high fat (Supplementary Fig 8a–c). Inhibition of either input did not lead to the development of a preference or aversion for the paired chamber (Supplementary Fig. 8d–g). Finally, given that chow is less appetitive than high fat and inhibition of either input did not affect normal chow intake we examined homeostatic feeding. Mice were food deprived for 36 h and thus highly motivated to consume chow (Supplementary Fig. 9a). Despite elevated levels of chow intake during a 20 min exposure, optogenetic inhibition of either input did not alter intake compared to controls (Supplementary Fig. 9b, c).

To further examine how modulation of aPVT and PFC inputs to NAc influences hedonic feeding, we used an operant behavioral task, which allows estimates of an individual’s motivation to work for a reward^24^. Sated mice previously exposed to high fat were trained on a fixed ratio 1 (FR1) schedule, where one nosepoke delivered a high fat pellet, and then on an FR5 schedule (Supplementary Fig. 10a). On days 17 and 18, mice underwent a progressive ratio (PR) test (Supplementary Fig. 10a), during which the number of pokes necessary for high fat delivery increased with each pellet^25^. Sated animals expressing either mCherry or hM4Di in NAc projecting aPVT neurons (Supplementary Fig. 10b) both increased responding to the same degree during the FR5 task (Supplementary Fig. 10c), demonstrating the high reward value of the high fat pellet. Administration of CNO 30 min prior to the PR, however, decreased the point at which responding ceases (that is, the breakpoint) in the hM4Di animals but not the mCherry animals when compared to the saline control injection (Supplementary Fig. 10d). When the same two-virus intersectional strategy was used to express the transgenes in NAc projecting PFC neurons (Supplementary Fig. 10e), again both groups of animals increased responding during the FR5 task (Supplementary Fig. 10f). However, CNO-triggered inhibition increased the breakpoint in hM4Di animals (Supplementary Fig. 10g). Together these results provide strong evidence that manipulations of aPVT and PFC inputs to NAc differentially regulate the motivation for, and/or, the hedonic value of high fat.

### Input-specific changes in synaptic strength following exposure to high fat

**Fig. 5:**
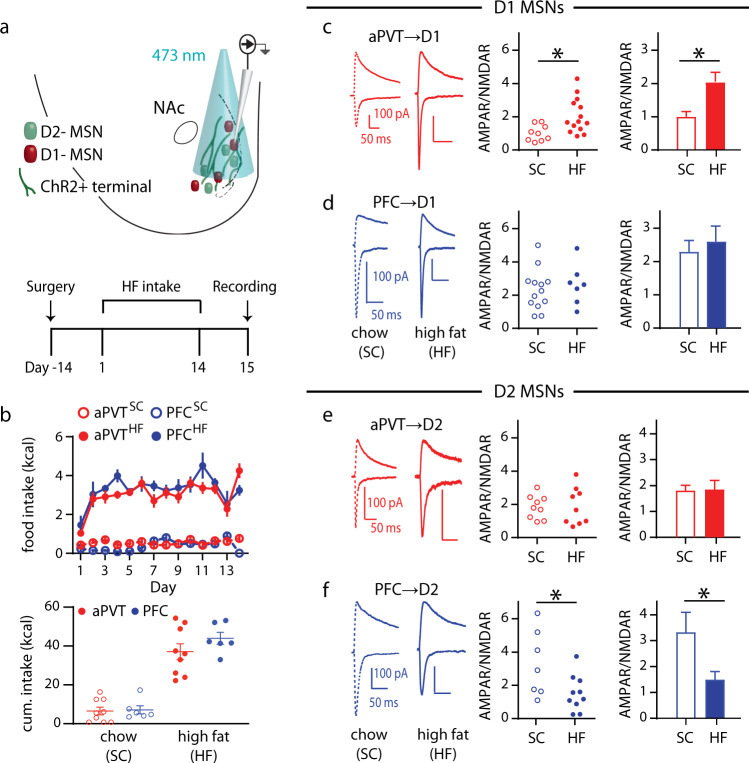
Chronic high fat intake modifies MSN synaptic strength in an input- and cell type-specific manner. **a** Schematic of experimental setup (top); experimental timeline (bottom). **b** (top) Daily intake of standard chow (SC) or high fat (HF) for mice expressing ChR2 in the PFC or aPVT; (bottom) Quantification of cumulative limited-access intake for PFC (n = 6/group) and aPVT mice (*n* = 9/group). **c**, **d** Representative traces of mice exposed to SC versus HF (left). Quantification of AMPAR/NMDAR ratio in D1 MSNs for (**c**) aPVT (*t* = 2.77, *P* = 0.01, *n* = 9, 14 cells/group, red) and (**d**) PFC (*n* = 13, 7 cells/group, blue) inputs. **e**, **f** Representative traces of mice exposed to SC versus HF (left). Quantification of AMPAR/NMDAR ratio in D2 MSNs for (**e**) aPVT (*n* = 9 cells/group, red) and (**f**) PFC (*t* = 2.37, *P* = 0.03, *n* = 7, 10 cells/group, blue) inputs. Data are ±s.e.m. *P < 0.05, Student’s *t* test two-tailed. Source data are provided as a Source Data file.

Given the ability of aPVT → NAc and PFC → NAc inputs to regulate high fat intake, we next examined if repeated intake of high fat leads to changes in the strength of these synapses, as observed following stress and administration of drugs of abuse^4–6,26,27^. ChR2 was injected into either the aPVT or PFC of D1-tdTomato/D2-eGFP transgenic mice^28^ and coronal slices were prepared for recording 24 h following the 14th day of limited-access exposure to high fat or standard chow (Fig. 5a). The ratio of AMPAR-mediated excitatory postsynaptic currents (EPSCs) to NMDAR-mediated EPSCs (AMPAR/NMDAR ratio) was used as a surrogate measure of synaptic strength^29^. Intake of high fat or standard chow was similar across the different input groups (Fig. 5b). At aPVT→D1 MSN synapses, there was a near doubling of the AMPAR/NMDAR ratio, while no significant differences occurred at PFC → D1 MSN synapses (Fig. 5c, d). Recording from D2 MSNs revealed no change in AMPAR/NMDAR ratios at aPVT→D2 MSN synapses as a consequence of high fat intake but a significant decrease in this ratio occurred at PFC → D2 MSN synapses (Fig. 5e, f). No differences were detected in the NMDAR EPSC decay kinetics in any condition, suggesting the AMPAR/NMDAR changes were due to alterations in AMPAR levels (Supplementary Fig. 11). The magnitude of the AMPAR/NMDAR ratios at PVT → D1 MSN synapses correlated with total high fat intake over the 2 weeks of exposure whereas this measure at PFC → D2 MSN inversely correlated with high fat intake (Supplementary Fig. 12).

### Depotentiation of NAc inputs alters high fat intake

**Fig. 6:**
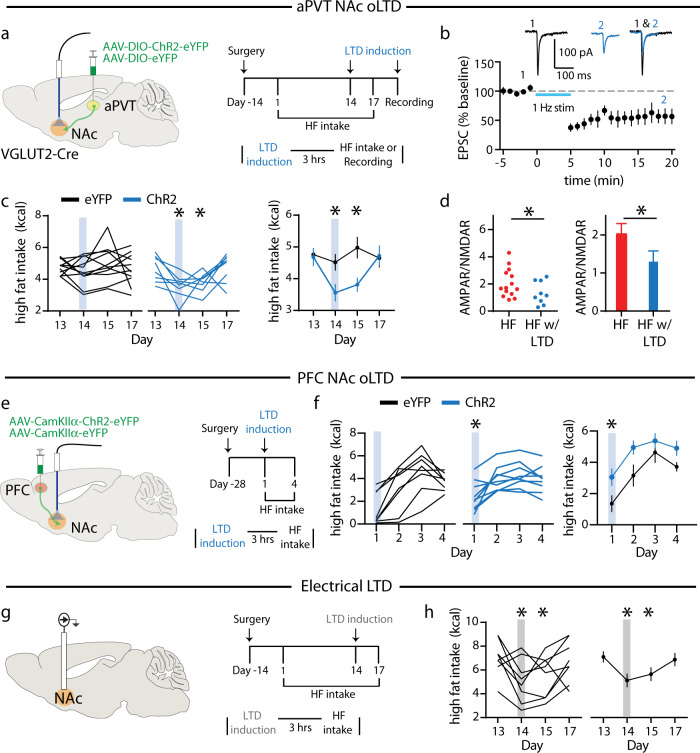
Inducing LTD at excitatory inputs to NAc regulates high fat intake. **a** Schematic of viral infection and ferrule implant for aPVT input stimulation (left); experimental timeline (right). **b** Summary time course and representative traces of EPSC amplitude demonstrating optically induced depression of aPVT→ D1 MSN synapses (*n* = 4 cells). **c** Quantification of high fat intake following LTD induction (*F*_3,51_ = 3.07, *P* = 0.03, *n* = 11, 8 mice/group) in eYFP control (black) and ChR2 expressing (blue) mice. **d** Quantification of AMPAR/NMDAR ratio in D1 MSNs for aPVT inputs exposed to high fat without (red) and with (blue) LTD induction (*t* = 1.75, *P* = 0.04, *n* = 14,9 cells/group). (Data for high fat group (red) in **d** are the same as those shown in Fig. 5c.) **e** Schematic of viral infection and ferrule implant for PFC input stimulation (left); experimental timeline (right). **f** Quantification of high fat intake following LTD induction (*F*_1,15_ = 4.57, *P* = 0.04, *n* = 8, 9 mice/group) in eYFP control (black) and ChR2 expressing (blue) mice. **g** Schematic of implant for electrical stimulation (left); experimental timeline (right). **h** Quantification of high fat intake following LTD induction (*F*_3,24_ = 4.59, *P* = 0.03, *n* = 9 mice). Gray bars signify electrical stimulation. Data are ±s.e.m. *P < 0.05, **b** Student’s *t* test two-tailed, **c**, **f** repeated measure two-way ANOVA with Sidak’s multiple comparison test, **d** Student’s *t* test one-tailed, **h** one-way ANOVA with Holm-Sidak multiple comparison test. Source data are provided as a Source Data file.

Do the input-specific changes in AMPAR/NMDAR ratios due to high fat intake contribute to the observed increases in hedonic feeding behavior? To address this question, we reversed the increase in AMPAR/NMDAR ratios at aPVT → D1 MSN synapses, 3 h before high fat exposure on day 14, by performing an optical LTD (oLTD) induction protocol (1 Hz stimulation for 15 min) of aPVT→NAc inputs in vivo^15^ (Fig. 6a), the efficacy of which was first confirmed by applying this protocol while recording EPSCs from tdTomato-expressing NAc D1 MSNs in acute slices (Fig. 6b). Following oLTD induction in vivo, high fat intake decreased on days 14 and 15, returning to baseline on day 17 (Fig. 6c). The efficacy of this in vivo oLTD induction protocol was then tested by applying a second round of optogenetic stimulation 3 h prior to sacrifice and then preparing acute brain slices from which AMPAR/NMDAR ratios were measured. This protocol reduced the AMPAR/NMDAR ratios at aPVT → D1 MSN synapses (Fig. 6d), thereby confirming the efficacy of the optogenetic stimulation in reducing synaptic strength.

To mimic the depression in strength at PFC → D2 MSN synapses following chronic high fat intake, 3 h before the first day high fat exposure, we performed an oLTD induction protocol of PFC → NAc inputs in vivo (Fig. 6e), similar to previous reports demonstrating that low frequency stimulation of this input can regulate reward-related behaviors^30–32^. The efficacy of this procedure was again confirmed by applying this protocol while recording EPSCs from tdTomato-expressing NAc D1 MSNs in acute slices (Supplementary Fig. 13). Following oLTD induction in vivo, high fat intake was significantly increased on day 1, and remained elevated compared to eYFP controls for several days (Fig. 6f). These results suggest that reduced activity in this input may contribute to increased levels of high fat intake.

Electrical DBS of the NAc transiently suppresses high fat intake using the same limited-access model^9,33^. To test whether, similar to the effects of oLTD at specific NAc inputs, applying an electrical non-specific LTD induction protocol might influence high fat intake, we implanted electrodes bilaterally into the NAc and again delivered a 1-Hz stimulation for 15 min, 3 h prior to high fat exposure on day 14 (Fig. 6g). High fat intake was reduced on days 14 and 15 but returned to baseline values on day 17 (Fig. 6h), similar to the effects of oLTD of aPVT → NAc inputs (Fig. 6c).

## Discussion

A long history of pharmacological manipulations of numerous neurotransmitter receptors in the NAc have demonstrated its critical role in regulating feeding behavior but have generated conflicting findings^4,21,22,34–36^, likely because such approaches cannot independently influence specific circuit elements that utilize the same neurotransmitter. For example, gross inhibition of NAc activity, via infusion of AMPA receptor antagonists or GABA agonists into the NAc shell, but not core, rapidly increased feeding^7^. However, a sustained increase in feeding occurs with AMPA infusion into the NAc in a dose-dependent manner, which was blocked by pretreatment with the opioid antagonist naltrexone^35^. Modern techniques, in particular optogenetics, facilitate more specific manipulations of NAc-related circuitry and have been used extensively to elucidate the input- and cell type-specific synaptic adaptations in the NAc caused by drugs of abuse or stress^4–6,26,27,29–32,36^. However, these methods are just beginning to be applied to determine whether similar adaptations occur following exposure to potent natural rewards, such as palatable high fat food. Here, we address this topic by assessing the role of two specific sets of excitatory inputs to NAc in high fat intake. Surprisingly, we find that manipulation of the activity of NAc inputs from aPVT and PFC influences high fat intake in an opposite manner. PFC input activity suppresses high fat intake while aPVT input activity promotes high fat intake. We also find unique adaptations at the NAc MSN synapses made by these inputs following prolonged high fat intake. Importantly, reversing the high fat-induced potentiation of aPVT → D1 MSN synapses using optogenetic stimulation in vivo caused a prolonged reduction in high fat intake, whereas mimicking the observed depression at PFC inputs increased initial intake.

Previous work examining the global adaptations at unknown sets of excitatory synapses on NAc MSNs due to dietary manipulations have produced confusing results with both high caloric intake and dietary restriction producing superficially comparable adaptations^4,35–38^. Similarly, manipulations of the activity of D1 and D2 NAc MSNs cause inconsistent effects on food intake^39–41^. Our results begin to provide an explanation for this confusion in that dietary changes caused different input-specific synaptic adaptations, which ultimately may increase excitatory drive at D1 MSNs. Consistent with the proposal that D1 MSN activity drives rewarding consumption, chronic restraint stress causes a decrease in the AMPAR/NMDAR ratio at synapses on D1 MSNs, and a corresponding decrease in food intake, sucrose preference and body weight^42^. However, other studies report that licking for sucrose in food-restricted rats is correlated with a pause in the firing of a large portion of NAc neurons^43^ and that inhibition of D1 MSNs increases licking for a liquid fat solution, via a projection to the lateral hypothalamus (LH)^41^. This unique subset of D1 MSN synapses also undergoes endocannabinoid-dependent plasticity following acute food restriction or high fat intake^38^. Yet, food palatability appears to be encoded by activation of a subset of NAc neurons^44^. Variation in important experimental parameters such as the nature of consumption (chewing vs licking), the satiety state of the subjects, the subpopulation of D1 MSNs being studied, and/or other methodological considerations, such as opsin variant, likely contribute to these seemingly inconsistent results. Indeed, following distinct types of stressors, dopamine (DA) release in the NAc oppositely regulates behavior^45,46^.

The PVT has been hypothesized to regulate feeding behavior^47^, but its specific role has remained unclear. Global inhibition of the PVT increases intake of standard chow^23,48^ whereas its global activation via infusion of orexin receptor agonists elicits robust high fat intake^23^. These superficially contradictory results may be due to the fact that these manipulations influenced different PVT circuit elements. Alternatively, the palatability of the food being tested, or its salience may influence the specific role of the PVT in food intake, comparable to the differential patterns of NAc activity discussed above^23,49^. Similarly, there are conflicting reports on the effects of PVT → NAc circuit modulation on operant reward seeking^20,50,51^. Here we find that inhibition of the aPVT → NAc circuit both decreased preference for a high fat paired chamber as well as the breakpoint in a PR task with high fat as the reinforcer. These results strengthen the hypothesis that manipulation of aPVT → NAc inputs regulates the incentive motivational properties of high fat rather than modulating hunger or satiety per se and is consistent with the lack of an effect on standard chow intake likely due to its low incentive value.

The behavioral similarities between pathological hedonic feeding and drug addiction, notably the compulsive seeking of appetitive stimuli despite adverse consequences, have led to the suggestion that there are important commonalities in the circuit adaptations mediating the behaviors^3,4,35,36^. Indeed, there is increasing evidence that in human subjects the brain regions impacted by drugs of abuse have altered activity in response to palatable food, including the striatum and prefrontal cortex^3,4^. However, here we provide evidence that there may be important differences in the specific input and cell type synaptic adaptations caused by drugs of abuse versus high fat intake. Notably, prolonged withdrawal from cocaine leads to a potentiation of strength in D1 MSN synapses made by PFC and hippocampal inputs whereas a potentiation of PVT → D2 MSN synapses occurs following acute withdrawal from morphine^15,30^. The circuit mechanisms by which these distinct synaptic adaptations in presumably similar cell populations lead to different behavioral adaptations are unclear. One contributing factor may be that the MSNs within specific subregions of the NAc shell subserve different functions^52,53^. For example, the specific rostral-caudal anatomical location of specific excitatory inputs into the NAc shell may be an important variable influencing the role of these inputs in food consumption and responses to a sucrose reward^11^. This is consistent with our finding that the aPVT and PFC inputs have both distinct and overlapping regions of innervation. Finally, differences in the detailed synaptic connectivity of aPVT and PFC inputs within the same NAc subregions, such as connections with distinct interneuron populations, may contribute to their different behavioral effects^23,54–56^.

Despite the differences in the specific synapses at which drug experiences and high fat cause potentiation, reversing the relevant synaptic changes by applying input-specific depotentiating stimuli in vivo reduce or reverse the behavioral adaptations caused by the drug or food experience^15,30^. Importantly, we demonstrate here that reversing the potentiation or mimicking the depression observed at aPVT or PFC inputs, respectively, alters high fat intake in a sustained manner. Furthermore, we found that electrical stimulation in a pattern that induces LTD, which would not be expected to have input-specific effects, reduced high fat intake for 2 days following the stimulation. This finding suggests that a neuromodulation strategy that does not require continuous stimulation nor input specificity may be a viable therapeutic intervention for pathological reward seeking in treatment-resistant populations.

## Methods

### Animals

Male C57Bl/6J were purchased from The Jackson Laboratory at 6-8 weeks of age. All transgenic mice (Slc17a6tm2(cre)Lowl/J; “VGLUT2-Cre”, Jackson Laboratory), B6.Cg-Tg(Drd1a-tdTomato)6Calak/J; “D1-tdTomato”, Jackson Laboratory), and B6;FVB-Tg(Drd2-EGFP/Rpl10a)CP101Htz/J; “D2-eGFP”, Jackson Laboratory) were bred inhouse on a C57Bl/6J background. Mice were individually housed on a 12-h light/dark schedule in a vivarium maintained at 71° Fahrenheit and 40% humidity with food and water ad libitum unless otherwise noted. House chow contained 28% protein, 60% carbohydrates, and 12% fat by calories (4.00 kcal/g: Purina Lab Diet). The high fat diet used to model binge-eating contained 20% protein, 20% carbohydrates, and 60% fat by calories (5.24 kcal/g: Research Diets). For operant studies, 60% fat Dustless Precision Pellets (14 mg; 4.95 kcal/g: Bio-Serv) were used. All procedures complied with the animal care standards set forth by the National Institute of Health and were approved by Stanford University’s Administrative Panel on Laboratory Animal Care and Administrative Panel of Biosafety. No statistical methods were used to predetermine sample size.

### Blinding

All experiments were conducted in a blinded manner such that assays were conducted and analyzed without knowledge of the specific manipulation being performed and with animals being randomized by cage before surgery and behavioral experiments. No animals were excluded from analysis for behavioral results except as noted below for CPP and operant assays

### Surgery

After 1 week of habituation to our facility, mice were anesthetized with a ketamine (100 mg/kg)-medetomidine (1 mg/kg) mixture, mounted in a stereotaxic frame and the skull surface was exposed. Thirty-three gauge syringe needles (Hamilton) were used to infuse 0.3 μl of virus into the region of interest (bregma coordinates: aPVT - anteroposterior −0.8, mediolateral 0.05, dorsoventral −3.2; mPFC – angle 10°, anteroposterior 1.7, mediolateral ± 0.8, dorsoventral −2.4; NAc – angle 10°, anteroposterior 1.6, mediolateral ± 1.5, dorsoventral −4.4) at a rate of 0.1 μl/min. Needles were removed 5 min after infusions were complete.

For optogenetic behavioral experiments, optic fibers (ferrules) were implanted above the NAc bilaterally (bregma coordinates: angle 10°, anteroposterior 1.6, mediolateral ±1.5, dorsoventral −4.2) for input stimulation. Ferrules were made in-house using 1.25 mm diameter multimode ceramic ferrules (ThorLab), 200 μm fiber optic cable with numerical aperture (NA) 0.39 (ThorLab) and blue dye epoxy (Fiber Instrument Sales). Ferrules were secured to the skull using miniature screws (thread size 00–90 × 1/16, Antrin Miniature Specialties) and light-cured dental adhesive cement (Geristore A&B paste, DenMat). For electrical stimulation in vivo, custom multielectrode arrays (70/30% Pt/Ir, 125 um; Microprobes) were implanted into NAc, according to the following coordinates relative to bregma: 1.34 mm anterior, 0.60 mm lateral, and 4.25 mm deep to brain surface.

### Viruses

Adeno-associated viruses (AAV) used in this study were purchased from the Stanford Neuroscience Gene Vector and Virus Core and included: AAV-DJ-ef1α-DIO-ChR2(H134R)-eYFP, AAV-DJ-ef1α-DIO-eNphR3.0-eYFP, AAV-DJ-ef1α-DIO-eYFP, AAV-DJ-CaMKIIa-ChR2(H134R)-eYFP, AAV DJ-CaMKIIa hChR2 (E123T/T159C)-p2A-EYFP-WPRE, AAV-DJ-CaMKIIa-eNphR3.0-eYFP, AAV-DJ-CaMKIIa-eYFP, AAV-DJ-CaMKIIa-mCherry, AAV-retro-CMV-hrGFP, AAV-DJ-ef1α-DIO-hM4D(Gi)-mCherry, and AAV-DJ-ef1α-DIO-mCherry. AAV titers ranged from 3.8 × 1012 to 1.7 × 1013 gc/m. The DJ serotype was used for its quick expression patterns allowing for input stimulation following 4 weeks of expression.

### High fat intake

Mice were singly housed 1 week prior to the onset of high fat intake. High fat chow was administered as previously described^9^. In brief, weight-matched mice were randomly assigned to each experimental condition. A single, pre-weighed high fat pellet was provided to the mice in their home cage daily for 1 hour at the same time each day for a given experiment. Intake of the high fat diet within that 1 h period was measured. For all electrophysiology experiments mice were fed high fat daily for 2 weeks. For optogenetic behavioral experiments, high fat was administered for 12 days prior to optogenetic manipulations. For experiments where acquisition of binge behavior was assessed, mice were exposed to high fat for 20 min, followed by 3 days of 20 min exposure to high fat with optogenetic stimulation. All mice with appropriate expression and targeting were included in data presentation and analyses.

### Conditioned place preference assays

The high fat CPP protocol was modified from standard protocols and conducted in an unbiased manner. Mice were placed in a 70 cm × 24 cm rectangular Plexiglass arena with three chambers that could be separated by removable Plexiglass walls. The left and right chambers each measured 28 × 24 cm and had distinct wall patterns (black and white stripes versus white with black circles) and flooring (smooth versus rough plastic floors). The center chamber measured 11.5 × 24 cm with no wall patterns and a smooth clear floor. On day 1, a baseline preference was conducted. Subjects were placed in the center compartment for 2 min at which point the barriers were lifted and the subject mouse was allowed to freely explore the entire apparatus for 30 min. On days 2–4, two conditioning session were conducted per day, separated by at least 3 hours. On days 2 and 4, in the first session the mouse was confined to one chamber that contained a high fat pellet with yellow light stimulation for 30 min; in the second session, the mouse was attached to the optical cables and confined to the opposite chamber, which was empty and without yellow light stimulation, for 30 min. On day 3, the order of the sessions was reversed to account for any confounds due to time of conditioning. High fat and stimulation paired side were counterbalanced. Mice that had a pre-test preference of less than 0.3 or greater than 3 were excluded (4 mice of 35 tested). The post-conditioning test was conducted in the same manner as the baseline preference test. The same protocol was used for the CPP experiments without a high fat pellet in the paired chamber and new distinct wall patterns (cross-hatched stripes and stars). Video tracking software (Viewer v3.0.1.442, BIOBSERVE) recorded all animal movements and automatically analyzed time spent and distance moved in each chamber. CPP score was calculated as: (time in high fat side_post_—time in high fat side_pre_).

### Motivated chow intake

On day 1, all chow was removed from the home cage before the dark cycle and mice were food deprived for 36 h. On the morning of day 3, mice were connected to the optical cables in their home cage. A pre-weighed chow pellet was placed in the home cage for 20 min, yellow light stimulation was delivered, and intake was measured for that period. All mice were included in data presentation and analyses.

### Operant progressive ratio task

Mice underwent a modified version of a progressive ratio task for high fat as previously described^24^. All mice had ad libitum access to chow throughout the experiment and were exposed to high fat for 2 weeks prior to training. Experiments were conducted in conditioning chambers (Med Associates Inc.) with a house light that turned on/off at the session start/end, two nosepoke ports that were illuminated and a food tray between the nosepoke ports in which the high fat pellet was delivered. Chambers were placed within sound-attenuating cubicles. Mice were first trained on a fixed ratio (FR)-1 schedule, where a single nosepoke in the active port turned off its light and allowed delivery of one high-fat pellet to the food tray. A 3-s time-out after each pellet delivery was included to allow time for mice to eat the pellets. Each mouse cohort had to average the delivery of at least 20 pellets per session before they were moved to an FR5 schedule, during which 5 nosepokes were necessary for pellet delivery. After 4 days of the FR5 schedule, the progressive ratio (PR) test occurred during which the number of pokes required to receive a pellet was *1* + *n*, with *n* = *pellets earned*. Groups were counterbalanced for injection of saline or CNO on PR test days. The final ratio completed was the breakpoint. Each session lasted for 1 hour or until 60 pellets were delivered. Four subjects (1 aPVT eYFP, 1 PFC eYFP, 2 PFC hM4Di) were excluded from analysis for failing to meet criteria during FR5 training.

### Optogenetic stimulation

For optogenetic photostimulation, ferrules were connected to a 473 nm laser diode (OEM Laser Systems or Laserglow Technologies) through a FC/PC adapter and a fiber optic rotary joint (Doric Lenses). Laser output was controlled using a Master-8 pulse stimulator (A.M.P.I.). Animals received either blue light (473 nm, 20 Hz, 5 ms pulse) or yellow light (532 nm, 8 sec on/2 s off) for the entire duration of high fat access using a 100-mW DPSS laser (532 nm - Shanghai Dream Lasers Technology Co, Ltd). For optical LTD experiments mice received blue light (473 nm, 1 Hz, 5 ms pulse) for 15 min, 3 h prior to high fat exposure. Intensity of light delivered to ferrule was ~10 mW (blue) or ~20 mW (yellow). For all in vivo optogenetic experiments mice were habituated to tethering for 20 min on the day prior to the first day of stimulation. At the completion of optogenetic experiments, histological analysis was performed to ascertain the level of virus expression and optical fiber placement. A small percentage of animals (<5%) were excluded based on the following criteria: (1) off-target transgene expression (clearly visualized substantial somatic expression of opsin outside the boundaries of the region of interest, either unilaterally or bilaterally), (2) weak transgene expression (clearly visualized low number of infected neurons, either unilaterally or bilaterally), (3) optical fiber placement was not in the medial NAc, i.e., was lateral of anterior commissure or tip was in the dorsal striatum, and/or (4) loss of headcap during the course of the experiment.

### Chemogenetic stimulation

Mice expressing either the inhibitory DREADD hM4Di or the mCherry control virus received CNO injections (10 mg/kg, Tocris) or equal volume of saline vehicle intraperitoneally ~30 min prior to the start of a limited-access exposure, PR operant task or 30 min prior to the beginning of the dark cycle for overnight measurements. Groups were counterbalanced so that half the mice received vehicle and the other CNO on a given day for operant experiments. All mice were habituated with a saline injection in the morning for the 2 days prior to experimental manipulations.

### Electrical LTD stimulation

Mice were tethered in their homecage and electrical stimulation (0.1 mA, 1 Hz, bi-polar, biphasic, 90 μs) was delivered for 15 min using a neural stimulation device (Alphalab SnR, Alpha Omega), 3 hours prior to high fat exposure.

### Electrophysiology

Whole-cell recordings were obtained from NAc D1-tdTomato or D2-eGFP medium spiny neurons in acute brain slices from mice that had been stereotaxically injected with ChR2 into the aPVT or PFC for synaptic physiology studies. All recordings occurred 24 h following day 14 of high fat exposure unless otherwise stated. To minimize stress and to obtain healthy slices, mice were anaesthetized with isofluorane and perfused immediately for 60 s with ice-cold artificial cerebrospinal fluid (aCSF), which contained in mM: 128 NaCl, 3 KCl, 1.25 NaH2PO4, 10 d-glucose, 25 NaHCO3, 2.5 CaCl2 and 2 MgCl2 (saturated with 95% O2 and 5% CO2, pH 7.4, 295–305 mOsm). Coronal acute brain slices containing the NAc were cut using a microslicer (VT1200 S, Leica) in cold sucrose aCSF which contained in mM: 254 sucrose, 3 KCl, 1.25 NaH2PO4, 10 d-glucose, 24 NaHCO3, 2 CaCl2, and 2 MgCl2 saturated with 95% O2 and 5% CO2. Slices were maintained in holding chambers with aCSF for 1 h at 32 °C. Patch pipettes (3–5 mΩ) for voltage-clamp recordings were filled with internal solution containing the following (mM): 117 cesium methanesulfonate, 2.8 NaCl, 0.4 EGTA, 5 tetraethylammonium chloride, 20 HEPES, 24 magnesium ATP and 0.4 GTP (pH 7.2, 285 mOsm). Whole-cell recordings were carried out using aCSF at 32 °C with the addition of 100 µM picrotoxin to block GABA-A receptor currents (flow rate = 2.5 ml/min). Postsynaptic recordings from medium spiny neurons in the shell were obtained under visual control using a 40× objective and made in areas of high ChR2 terminal infectivity while activating ChR2 with 473-nm blue light. The NAc shell was identified by the presence of the anterior commissure. Series resistance (10–30 MΩ) was monitored with a 5-mV hyperpolarizing pulse (10 ms) given during every stimulation and only recordings that remained stable (<20% change in series resistance) over the period of data collection were used.

To elicit input-specific excitatory postsynaptic currents (EPSCs), every 10 seconds, a 1-ms blue light pulse was delivered by an LED (ThorLabs) directed through the objective with an intensity of ~5 mW. Recordings were performed using a Multiclamp 700B (Molecular Devices), filtered at 4 kHz and digitized at 10 kHz. Data acquisition and analysis were performed using AxoGraph X v1.5.4. Cells were held at a holding potential of −70 mV to measure AMPAR-mediated EPSCs. AMPAR/NMDAR ratios were calculated by collecting 30 consecutive EPSCs at −70 mV and then depolarizing the cell to +40 mV and collecting another 30 consecutive EPSCs. The peak (2 ms window) of the averaged EPSC at −70 mV (n = 30) was used to calculate the AMPAR-mediated EPSC. The NMDAR-mediated EPSC was calculated by measuring the amplitude of the averaged EPSC at +40 mV (*n* = 30) 50–55 msec after the light pulse. To induce input-specific LTD at aPVT → NAc inputs in acute slices, optical EPCSs were recorded for 5 minutes at 0.1 Hz at which point 1 Hz-5 min optical stimulation was delivered. Summary LTD graphs were generated by averaging the peak amplitudes of individual EPSCs in 1-min bins (six consecutive sweeps) and normalizing these to the mean value of EPSCs collected during the 5 min baseline immediately before the LTD induction protocol. For in vivo input-specific optical LTD, 1 Hz-15 min stimulation was applied via the fiber optics in the NAc shell and acute coronal slices were prepared 3 hours later. All recordings and analyses were performed blinded, without knowledge of the experimental condition.

### Histological procedures and imaging

Animals were transcardially perfused with 4% paraformaldehyde (PFA) or 10% formalin and then further post-fixed overnight. Coronal sections (50 μM) were prepared on a vibratome in PBS. Sections were blocked with 10% normal donkey serum (NDS) and 2% bovine serum albumin (BSA) in PBS/0.5% triton-X100 (PBST), then incubated in primary antibody in 1% NDS in PBST overnight at room temperature (RT). Sections were rinsed three times for 30 minutes each at room temperature, followed by incubation in secondary antibody stain in 1% NDS in PBT at RT for 3 h. Sections were then rinsed three times (30 min/rinse), mounted on slides, coverslipped with Fluoromount-G mounting medium (Southern Biotech). Concentrations and sources for antibodies were as follows: chicken anti-GFP 1:2000 (Aves labs, GFP-1020), rabbit anti-cFos 1:500 (Synaptic Systems, 226003), rat anti-mCherry 1:1500 (Invitrogen, M11217), rabbit anti-GAD65 1:1000 (PA5-22260), and donkey anti-rabbit, chicken, or rat secondary corresponding antibodies 1:200 (Alexa Fluor 488, & 584). Image acquisition was performed with a Nikon A1 confocal system using a ×10 or ×20 objective. When quantitative or qualitative comparisons were made between images, all image acquisition and post-processing settings were held constant.

To quantify optogenetic regulation of neural activity, animals were euthanized, via transcardial perfusion, 90 min after optical stimulation at 20 Hz for 15 min, and histologically processed as noted above. Averages per animal were obtained by counting cFos+ nuclei within a defined anterior-posterior segment (5 serial sections ranging from 1.6 to 1.0 mm relative to bregma). Analysis was then conducted in a semi-automatic, blinded fashion using ImageJ v2.1.0/1.53 g (Fiji) in-house macros to automatically detect and count cFos+ nuclei. In brief, z-stack (4 µm steps) images were acquired at 10X and converted to max intensity projections (MIPs). Then the local background was subtracted from the MIP (rolling ball radius of 30 pixels), thresholded by the mean background multiplied by a factor of 2, and then binarized to a mask. The ImageJ Analyze Particles function was used to count cFos+ nuclei within each manually drawn ROI. Parameters were manually verified to most accurately count cFos+ nuclei in a random subset of images.

To quantify terminal innervation and colocalization, VGLUT2-Cre mice expressing distinct fluorophores in the aPVT and PFC (aPVT-DIO-eYFP:PFC-CamKIIa-tdTomato, *n* = 3; aPVT-DIO-mCherry:PFC-CamKIIa-mCherry, *n* = 2) were euthanized via transcardial perfusion and histologically processed as noted above. Analysis was then conducted in a semi-automatic, blinded fashion using ImageJ (Fiji) plugins and in-house macros to automatically detect and analyze axon colocalization. In brief, z-stack (4 µm steps) images were acquired at 20X and converted to max intensity projections (MIPs). Then the local background was subtracted from the MIP (rolling ball radius of 30 pixels). Using the resulting images, within defined anterior-posterior NAc subregions (three serial sections ranging from 1.42 to 1.0 mm relative to bregma), colocalization was analyzed using the Coloc2 function in ImageJ to obtain correlation coefficients. Correlation heatmaps were generated using the ImageJ Colocalization Colormap plugin.

### Statistics

As mentioned above, for all data acquisition and analyses in this study, investigators were blinded to the manipulation that the experimental subject had received and the genotype of the subject. Student’s *t* tests two-tailed were used when comparing only two groups unless otherwise stated. One-way sample t and Wilcoxon test was used to test whether the average correlation significantly deviated from zero. One-way ANOVA with Tukey’s post hoc test was used to compare multiple groups. Two-way ANOVA was used for analysis of multiple groups with Sidak’s, or Tukey’s multiple comparison post hoc test, when appropriate. Statistical analyses were performed using Prism 9.0 (GraphPad Software). All data were tested and shown to exhibit normality and equal variances. All data are expressed as mean ± s.e.m.

### Reporting summary

Further information on research design is available in the Nature Research Reporting Summary linked to this article.

## Supplementary information

Supplementary Information

Reporting Summary

## Data Availability

All data are available from the corresponding author upon reasonable request.

## Source data

Source Data
