# Supplementary material for: Input-specific modulation of murine nucleus accumbens differentially regulates hedonic feeding
Source: Nat Commun. 2021 Apr 9;12:2135. doi: 10.1038/s41467-021-22430-7 (PMC8035198; doi:10.1038/s41467-021-22430-7)
Supplement: Supplementary file 1 — Supplementary Information [file 41467_2021_22430_MOESM1_ESM.pdf]

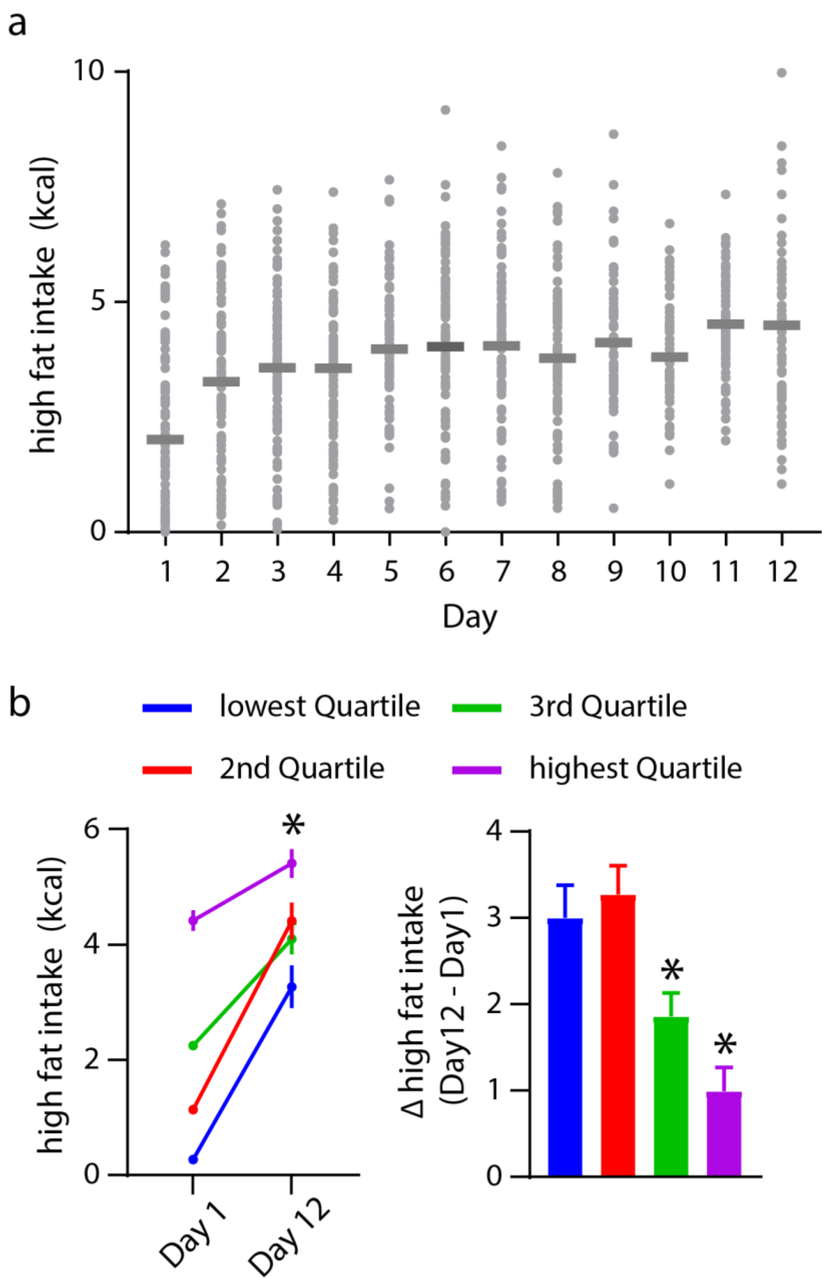

Supplementary Figure 1. Variability in high fat intake during daily one hour exposure.

a, Individual data points and mean distribution of high fat intake per exposure across days 1-12. **b,** High fat intake per exposure for lowest to highest quartile for day 1 and day 12 (left; $F_{3,108} = 11.1$, $P = 0.0001$, n = 28 mice/group) and the difference in intake levels between day 12 and day 1 (right; $F_{3,108} = 11.07$, $P = 0.0001$, n = 28 mice/group). Data are mean ± s.e.m. *$P < 0.05$, one-way ANOVA with Dunnet's multiple comparison post hoc test. Source data are provided as a Source Data file.

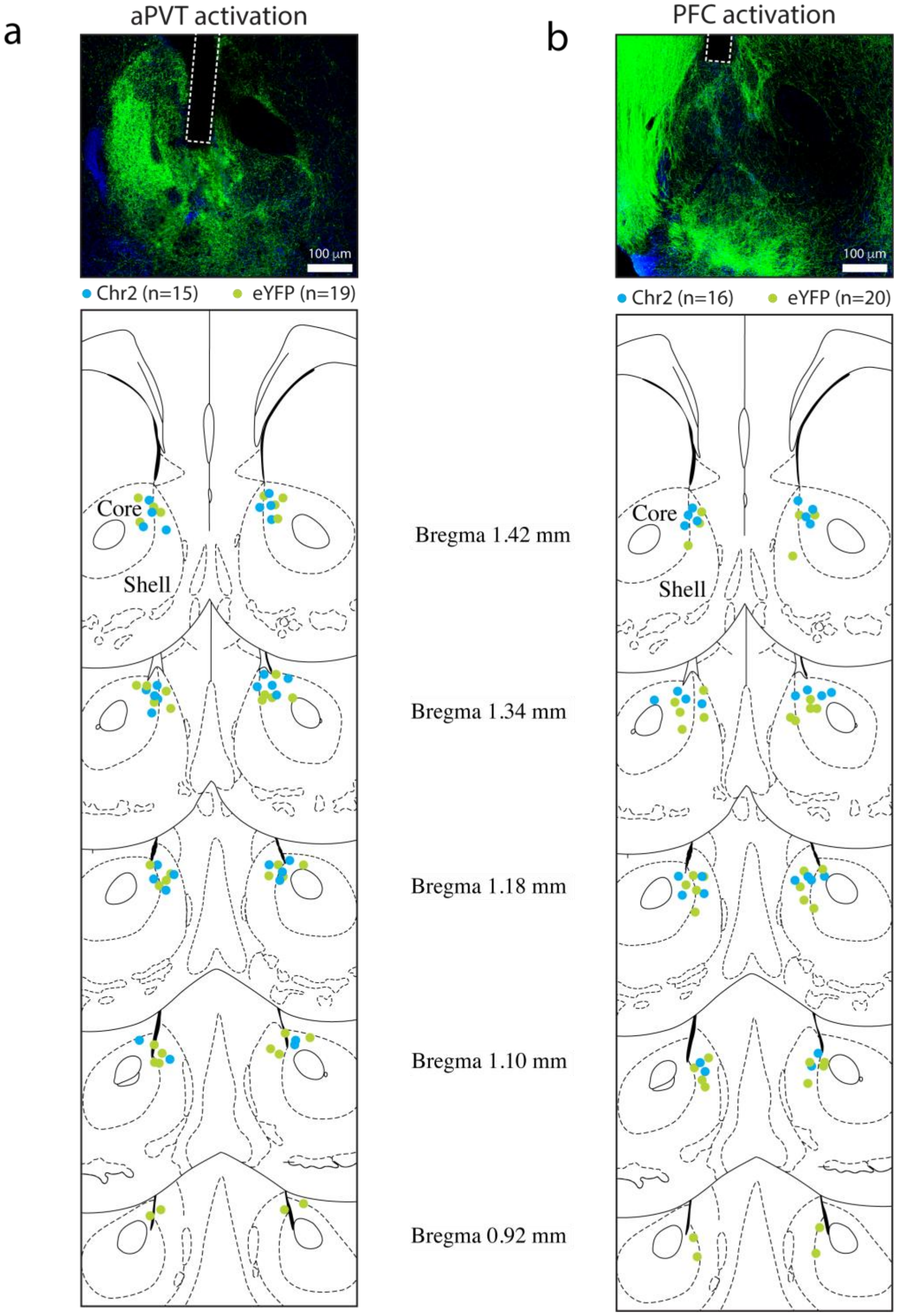

Supplementary Figure 2. Summary of histological assessment of optic fiber tip placement for activation studies. a, Representative images of transgene (green) expression and fiber optic placement for aPVT activation experiments (top); summary of optic fiber tip placement (blue = ChR2; green = eYFP). **b,** Representative images of transgene (green) expression and fiber optic placement for PFC activation experiments (top); summary of optic fiber tip placement (blue = ChR2; green = eYFP).

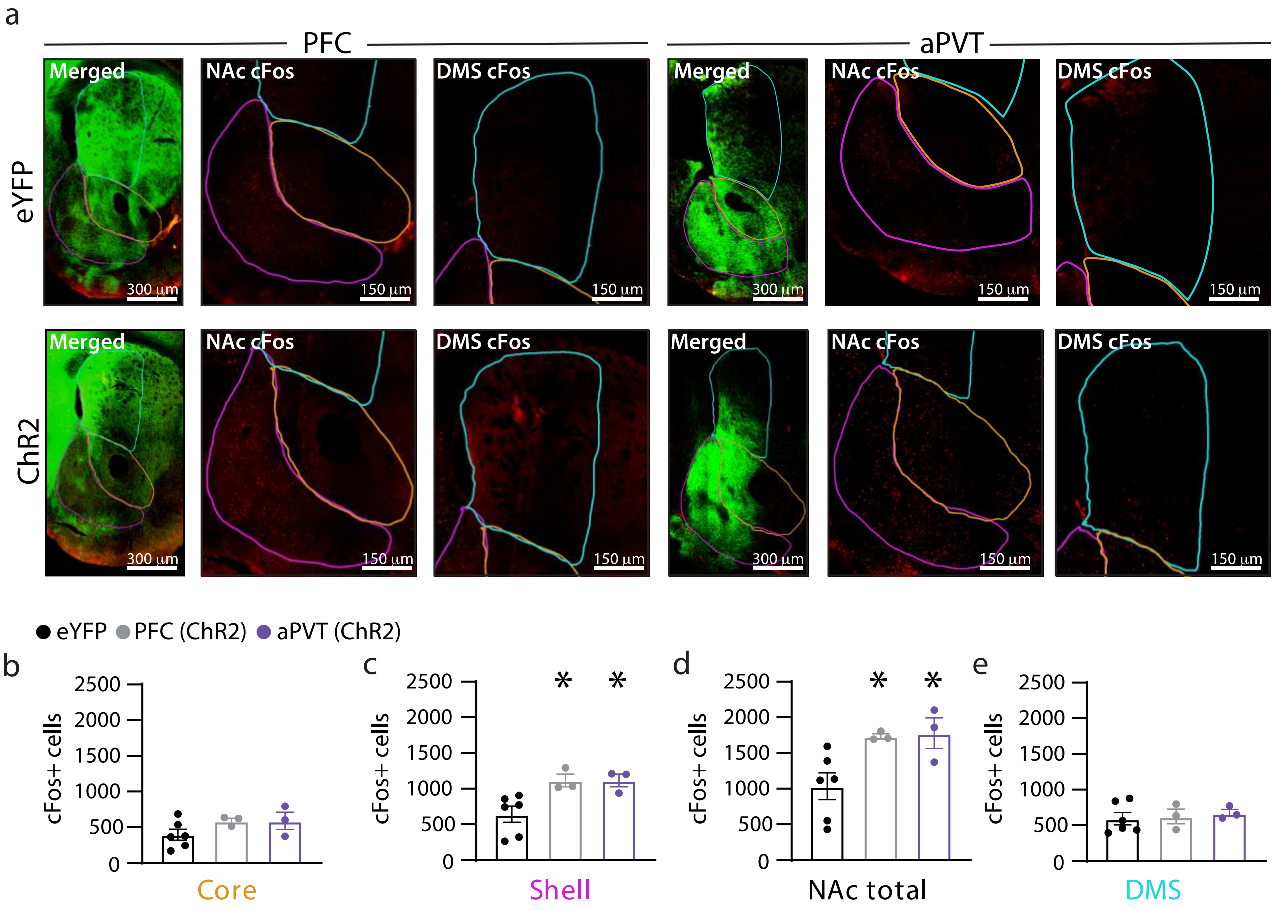

Supplementary Figure 3. Activation of distinct NAc glutamatergic inputs selectively increase cFos levels in the NAc.

a, Representative images of transgene (green) and cFos (red) (left most image), and magnified cFos only (middle, right) expression patterns for PFC-eYFP control (top-left), PFC-ChR2 (bottom-left), and aPVT-eYFP (top-right), aPVT-ChR2 (bottom-right) mice. **b-e,** Quantification of cFos+ cells in the NAc core (**b:** n = 6,3 mice/group), shell (**c:** $F_{2,9} = 6.25$, $P = 0.02$, n = 6,3 mice/group), total NAc (**d:** $F_{2,9} = 5.23$, $P = 0.03$, n = 6,3 mice/group), and medial dorsal striatum (DS) (**e:** n = 6,3 mice/group). Data are mean ± s.e.m. *P< 0.05, one-way ANOVA with Tukey's multiple comparison post hoc test. Source data are provided as a Source Data file.

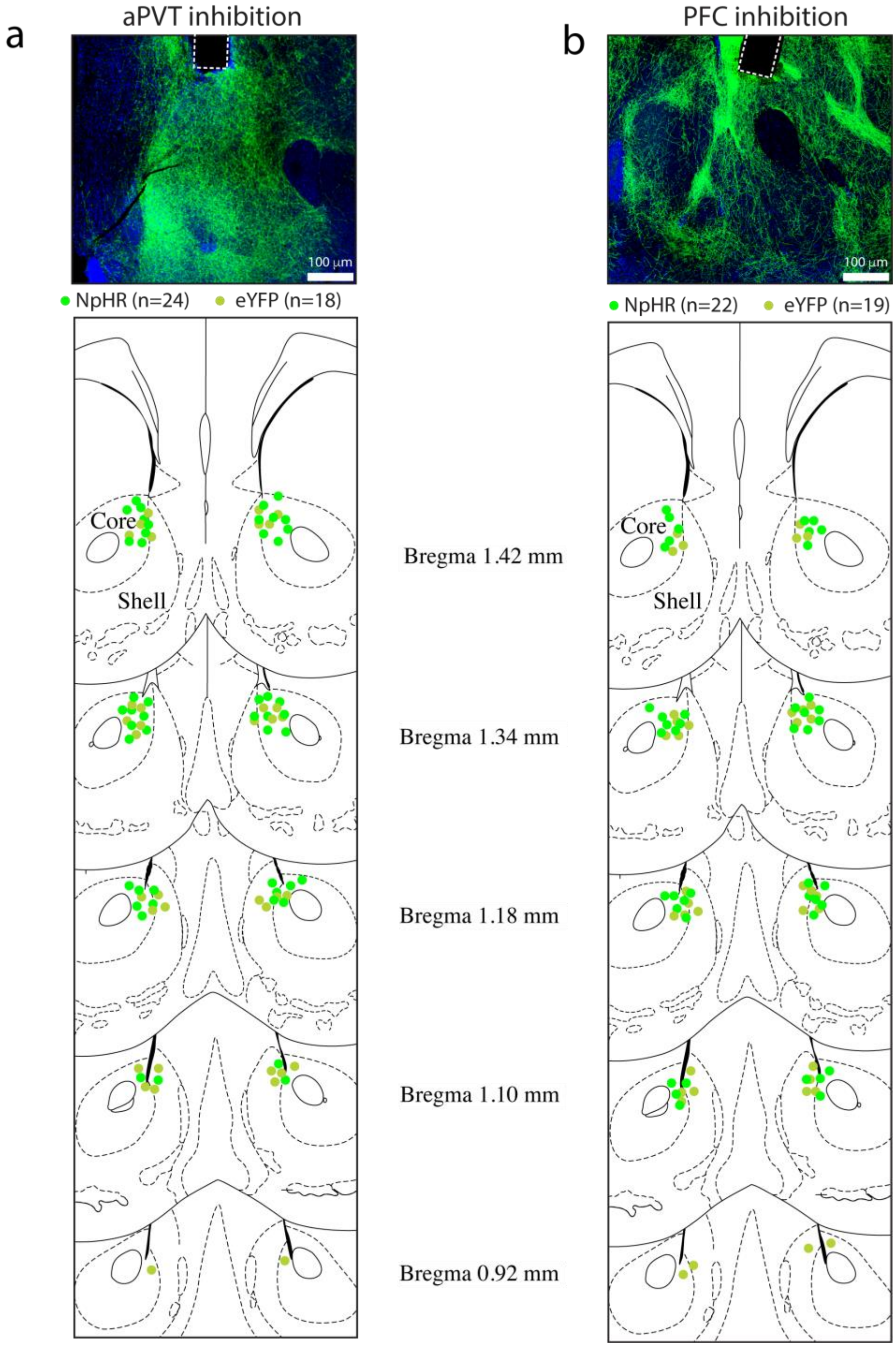

Supplementary Figure 4. Summary of histological assessment of optic fiber tip placement for inhibition studies. a, Representative images of transgene (green) expression and fiber optic placement for aPVT inhibition experiments (top); summary of optic fiber tip placement (bright green = NpHR; yellow-green = eYFP). **b,** Representative images of transgene (green) expression and fiber optic placement for PFC inhibition experiments (top); summary of optic fiber tip placement (bright green = NpHR; yellow-green = eYFP).

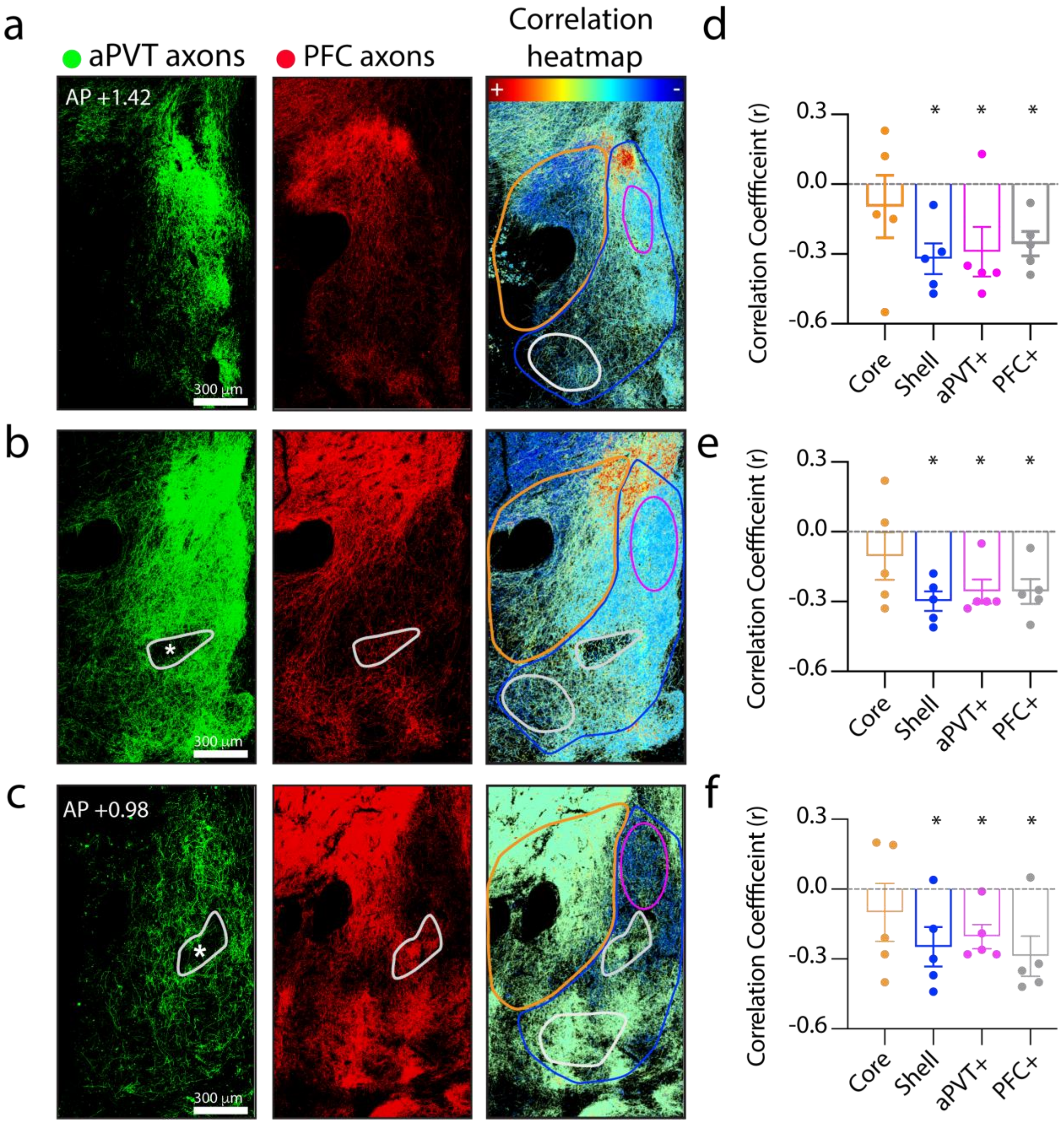

Supplementary Figure 5. Differential Innervation of NAc subregions by aPVT and PFC inputs. Representative images (from left to right) of aPVT axons, PFC axons, and correlation heatmap in NAc subregions at **a**) AP +1.42, **b**) AP +1.18, **c**) AP +0.98. **d-f**, Quantification of input colocalization with correlation coefficient (**d:** Core – t =0.08; Shell – t =2.99, P=0.008; aPVT+ – t=3.98, P=0.02; PFC+ – t=3.37, P=0.03 **e:** Core – t =0.02; Shell – t =7.12, P=0.002; aPVT+ – t=4.94, P+0.008; PFC+ – t=4.81, P=0.009 **f:** Core – t =0.80; Shell – t =2.93, P=0.04; aPVT+ – t=3.98, P=0.02; PFC+ – t=3.34, P=0.03; n = 5 mice/group) of NAc subregions for identified AP sections in a-c. Data are mean ± s.e.m. *P< 0.05, one-way sample t and Wilcoxon test. Source data are provided as a Source Data file.

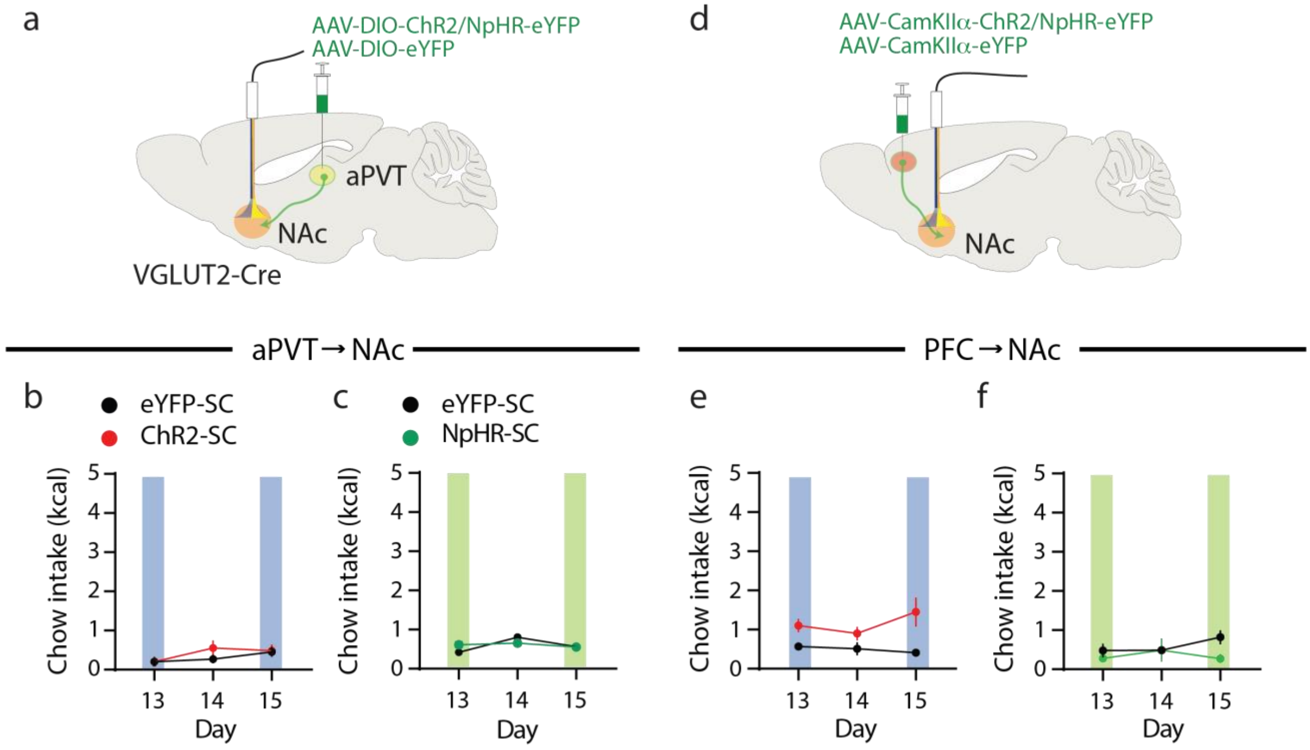

Supplementary Figure 6. Manipulation of distinct NAc glutamatergic input activity does not affect chow intake.

a, Schematic of viral injection and ferrule implant for aPVT input stimulation. **b,** Quantification of chow intake during expression period (n = 5,9 mice/group) in eYFP control (black) and ChR2 expressing (red) mice. Blue bars signify blue light stimulation. **c,** Quantification of chow intake during expression period (n = 12,8 mice/group) in eYFP control (black) and NpHR expressing (green) mice. Green bars signify green light stimulation. **d,** Schematic of viral injection and ferrule implant for PFC input stimulation. **e,** Quantification of chow intake during expression period (n = 5,6 mice/group) in eYFP control (black) and ChR2 expressing (red) mice. **f,** Quantification of chow intake during expression period ($P > 0.05$, n = 5 mice/group) in eYFP control (black) and NpHR expressing (green) mice. Data are mean ± s.e.m. two-way ANOVA with Sidak's multiple comparison post hoc test. Source data are provided as a Source Data file.

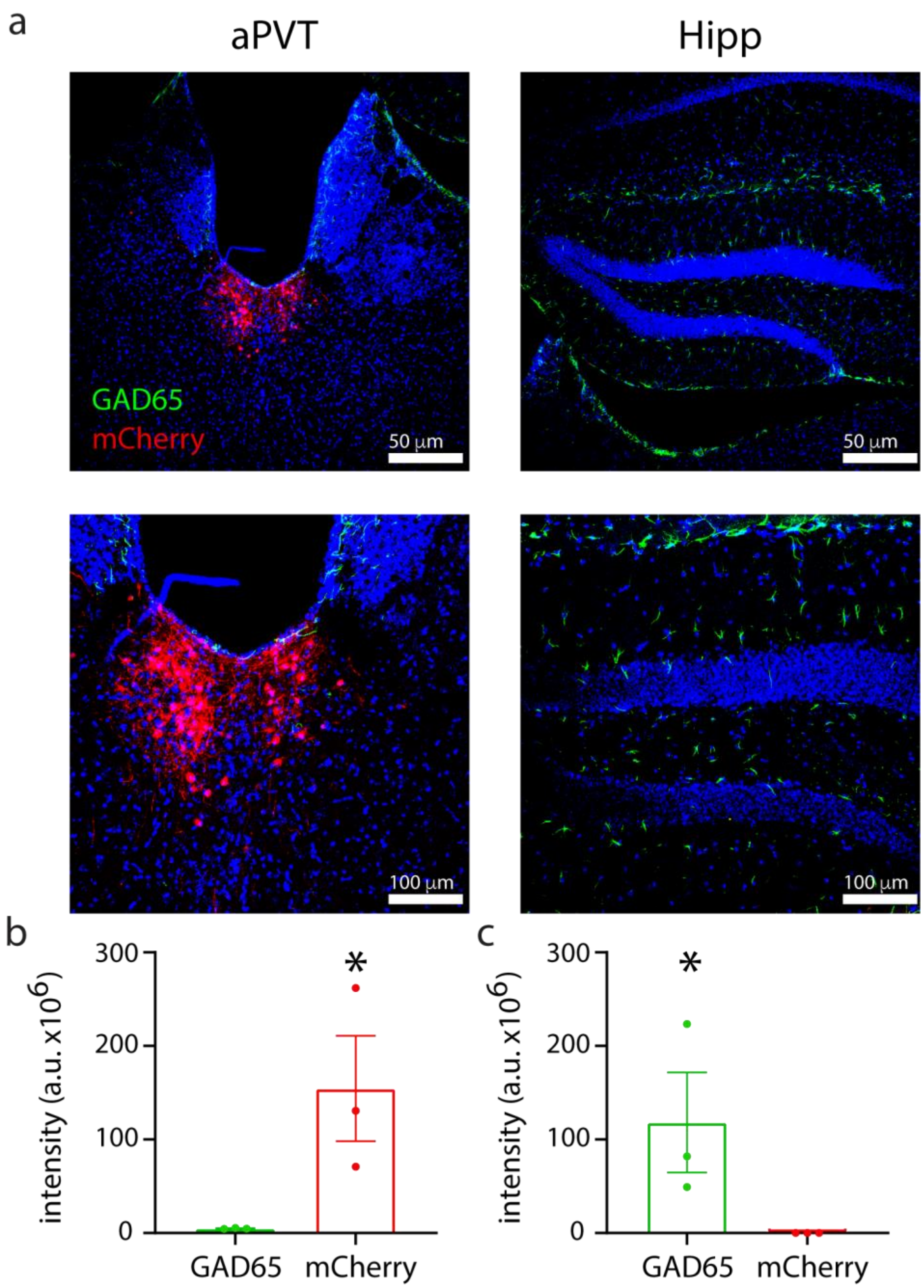

Supplementary Figure 7. NAc projecting PVT neurons are not GABAergic.

a, Representative images of GAD65 (green) and mCherry (red) (top 10x; bottom 20x) expression patterns in the aPVT and hippocampus (Hipp). **b, c** Quantification GAD65 and mCherry fluorescence in the aPVT (**b**: $t = 2.33$, $P < 0.01$, $n = 3$ mice/group) and Hipp (**c:** $t = 2.21$, $P < 0.05$, $n = 3$ mice/group) Statistical analysis of GAD65 versus mCherry expression for aPVT versus PFC (two-way ANOVA: $F_{1,8} = 10.2$, $P < 0.05$, $n = 3$ mice/group). Data are mean ± s.e.m. Source data are provided as a Source Data file.

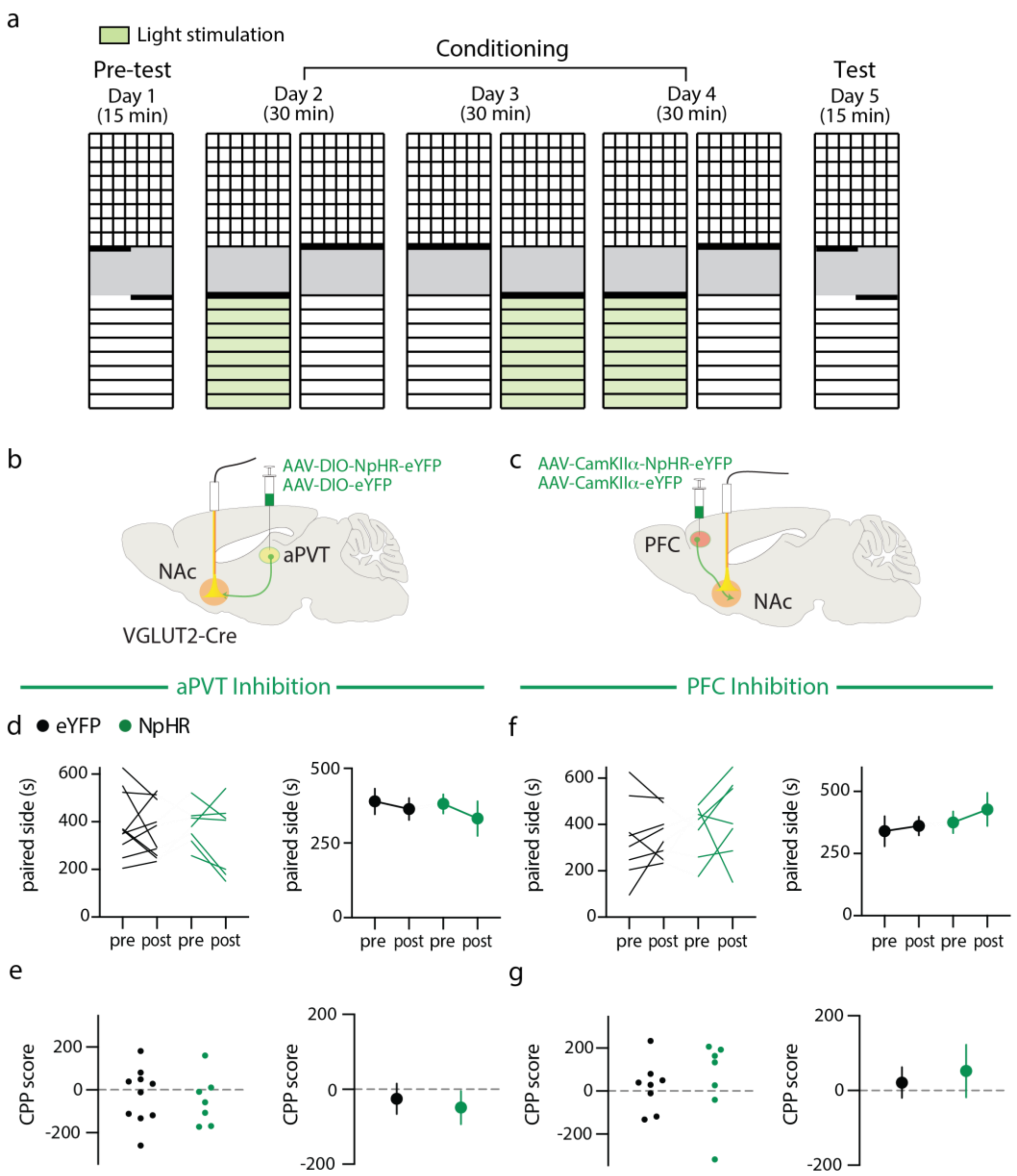

Supplementary Figure 8. Inhibition of distinct NAc glutamatergic inputs does not induce conditioned place preference or aversion.

a, Schematic of experimental setup and design. **b,** Schematic of viral injection and ferrule implant for aPVT input inhibition. **c,** Schematic of viral injection and ferrule implant for PFC input inhibition. **d, e,** Quantification of time spent in the paired chamber (**d**: n = 10,7 mice/group) and CPP score for paired chamber (**e**: n = 10,7 mice/group) in eYFP control (black) and NpHR expressing (green) mice. **f, g,** Quantification of time spent in paired chamber (**f**: n = 8,7 mice/group) and CPP score for paired chamber (**g**: n = 8,7 mice/group) in eYFP control (black) and NpHR expressing (green) mice. Data are mean ± s.e.m. (d, f) two-way ANOVA with Sidak's multiple comparison post hoc test, (e, g) student's t-test 2-tailed. Source data are provided as a Source Data file.

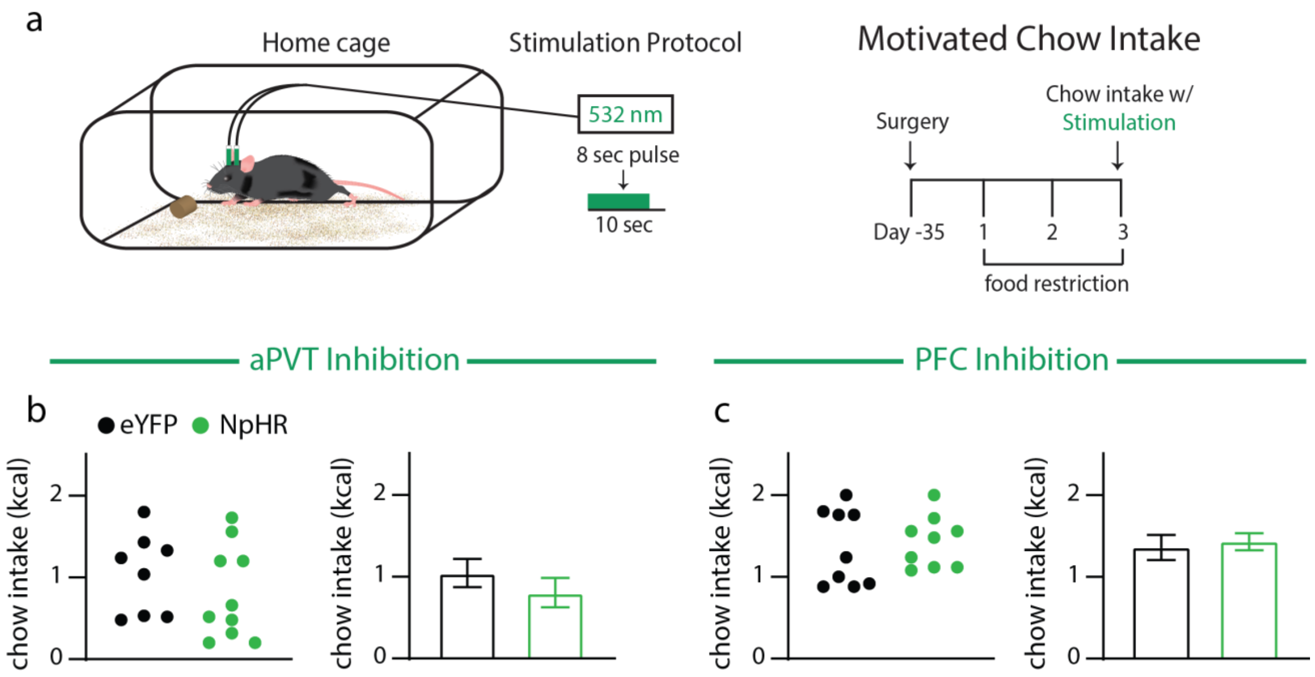

Supplementary Figure 9. Inhibition of distinct NAc glutamatergic inputs does not affect motivated chow intake.

a, Schematic of experimental setup (left); experimental timeline (right). **b, c,** Quantification of chow intake following food deprivation (**b**: n = 8,10 mice/group; **c:** n = 9 mice/group) in eYFP control (black), aPVT (**b**) and PFC (**c**) NpHR expressing (green) mice. Student's t-test 2-tailed. Data are mean ± s.e.m. Source data are provided as a Source Data file.

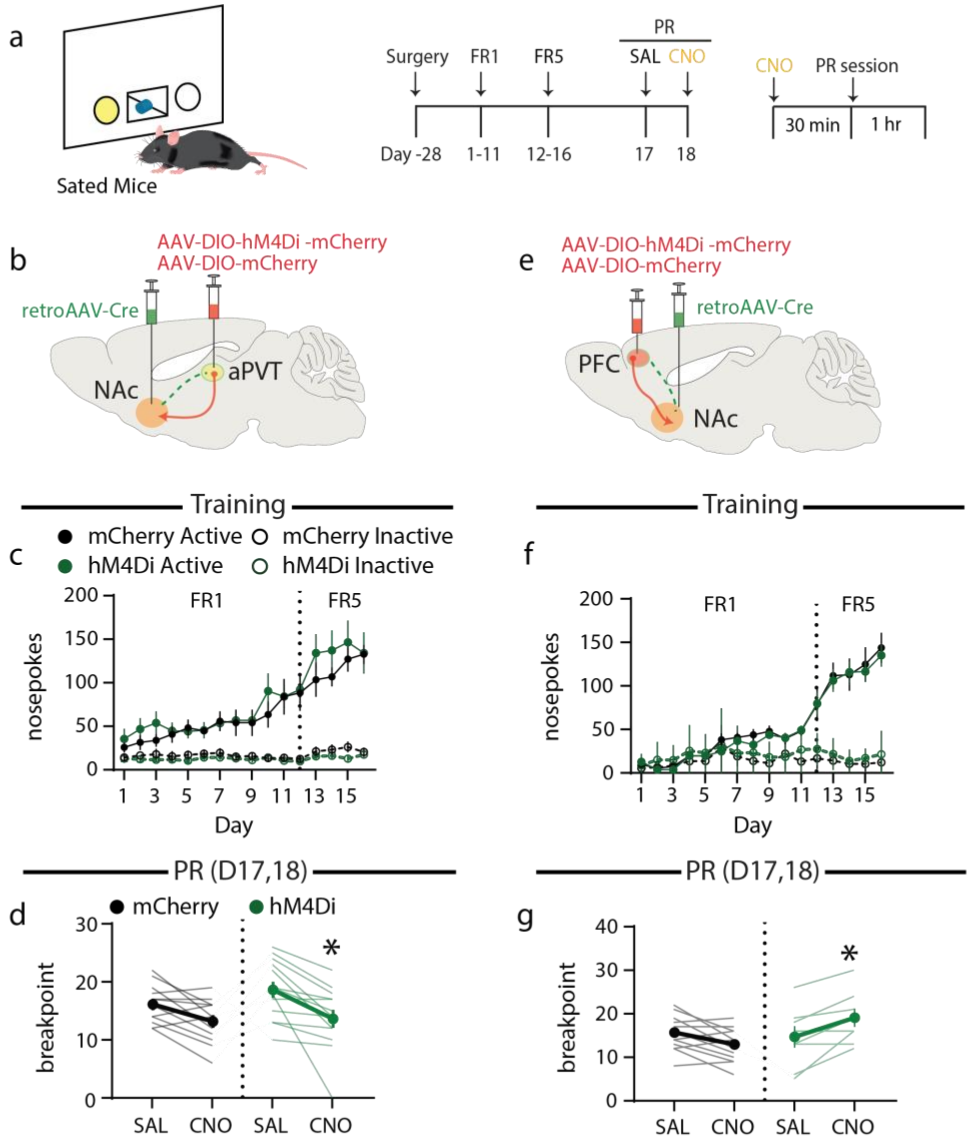

Supplementary Figure 10. Inhibition of distinct NAc projection neurons selectively regulates motivation for high fat.

a, Schematic of experimental setup (left); experimental timeline (right). **b,** Schematic of viral injection for NAc projecting aPVT neuron inhibition. **c,** Quantification of training demonstrating sated mice will nosepoke for high fat pellet: mCherry control (black) and hM4Di expressing (green) mice. **d,** Quantification of breakpoint (**d:** $F_{1,23} = 20.5$, $P = 0.0002$, **c,d:** n = 12,13 mice/group) in mCherry control (black) and hM4Di expressing (green) mice. **e,** Schematic of viral injection for NAc projecting PFC neuron inhibition. **f,** Quantification of training demonstrating sated mice will nosepoke for high fat pellet: mCherry control (black) and hM4Di expressing (green) mice. **g,** Quantification of breakpoint (**g:** $F_{1,18} = 17.8$, $P = 0.0005$, **f,g:** n = 12,8 mice/group) in mCherry control (black) and hM4Di expressing (green) mice. Data are mean ± s.e.m. *$P < 0.05$, two-way ANOVA with Sidak's multiple comparison post hoc test. Source data are provided as a Source Data file.

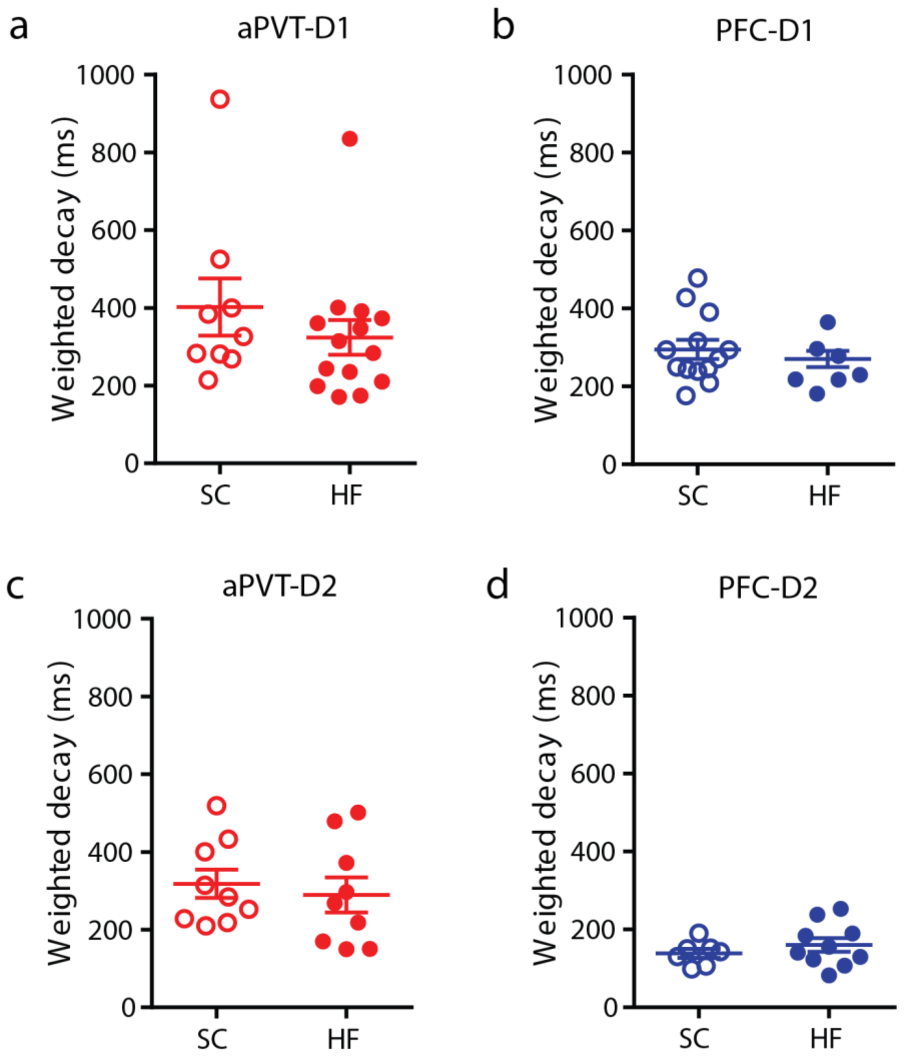

Supplementary Figure 11. High fat intake does not alter the decay kinetics of NMDAR EPSCs. Quantification of NMDAR EPSC weighted decay in D1 MSNs for **a,** aPVT (n = 9,14 cells/group, red) and **b**, PFC n = 13 cells/group, blue) inputs. Quantification of NMDAR EPSC weighted decay in D2 MSNs for **c,** aPVT (n = 9 cells/group, red) and **d**, PFC (n = 7,10 cells/group, blue) inputs. Data are mean ± s.e.m. Student's t-test, 2-tailed. Source data are provided as a Source Data file.

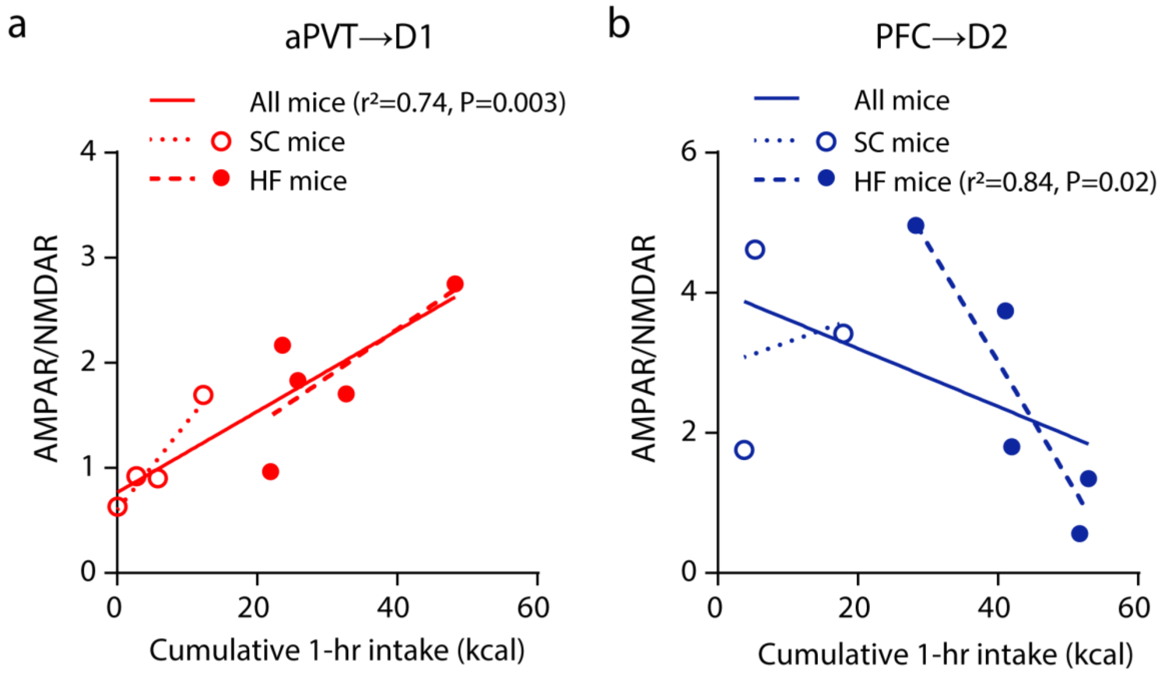

Supplementary Figure 12. Changes in synaptic strength correlate with cumulative intake.

a, Linear regression analysis revealing a significant correlation of the AMPAR/NMDAR ratio at aPVT→D1 MSN synapses with cumulative intake of chow and high fat during 1-hr exposure (r^2=0.74, P=0.003). **b,** Linear regression analysis revealing a significant inverse correlation of the AMPAR/NMDAR ratio at PFC→D2 MSN synapses with cumulative intake of high fat during 1-hr exposure (r^2=0.84, P=0.02). Source data are provided as a Source Data file.

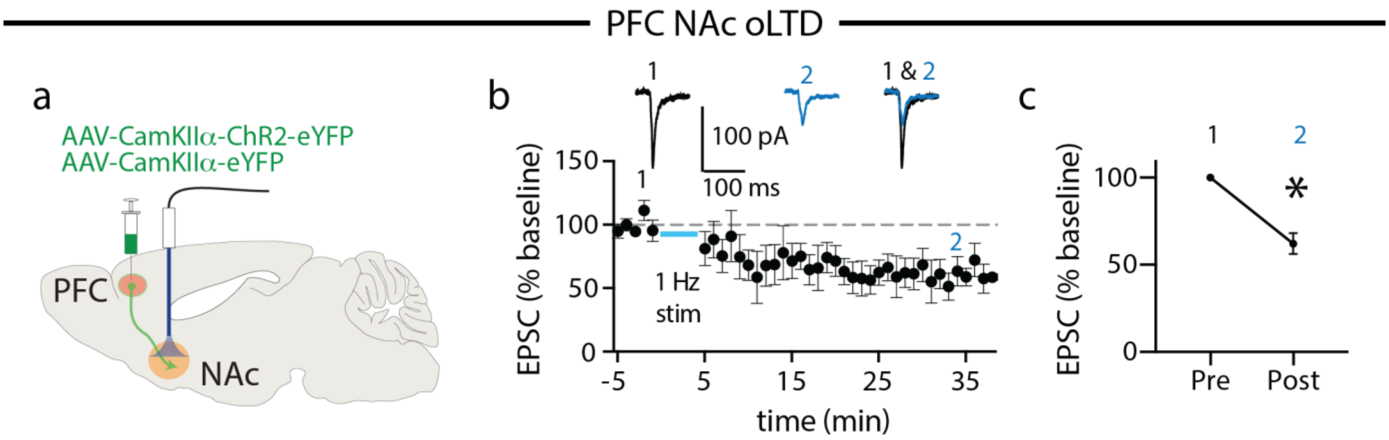

Supplementary Figure 13. Induction of LTD at PFC→NAc synapses.

a, Schematic of viral infection and ferrule implant for PFC input stimulation. **b,** Summary time course and representative traces of EPSC amplitude demonstrating optically induced depression of PFC→NAc synapses**. c,** Quantification of EPSC amplitude pre- and post-LTD induction protocol (**c**: t = 6.40, P = 0.007, **b,c:** n = 4 cells). Student's t-test 2-tailed. Data are mean ± s.e.m. *P< 0.05, student's t-test. Source data are provided as a Source Data file.
