# Supplementary material for: Input-specific modulation of murine nucleus accumbens differentially regulates hedonic feeding
Source: Nat Commun. 2021 Apr 9;12:2135. doi: 10.1038/s41467-021-22430-7 (PMC8035198; doi:10.1038/s41467-021-22430-7)
Supplement: Supplementary file 2 — Reporting Summary [file 41467_2021_22430_MOESM2_ESM.pdf]

nature research

Corresponding author(s): Robert Malenka

Last updated by author(s): 2/21/2021

Reporting Summary

Nature Research wishes to improve the reproducibility of the work that we publish. This form provides structure for consistency and transparency in reporting. For further information on Nature Research policies, see Authors & Referees and the Editorial Policy Checklist .

Statistics

For all statistical analyses, confirm that the following items are present in the figure legend, table legend, main text, or Methods section.

n/a	Confirmed	
☐	☒	The exact sample size (*n*) for each experimental group/condition, given as a discrete number and unit of measurement
☐	☒	A statement on whether measurements were taken from distinct samples or whether the same sample was measured repeatedly
☐	☒	The statistical test(s) used AND whether they are one- or two-sided<br>*Only common tests should be described solely by name; describe more complex techniques in the Methods section.*
☒	☐	A description of all covariates tested
☒	☐	A description of any assumptions or corrections, such as tests of normality and adjustment for multiple comparisons
☐	☒	A full description of the statistical parameters including central tendency (e.g. means) or other basic estimates (e.g. regression coefficient) AND variation (e.g. standard deviation) or associated estimates of uncertainty (e.g. confidence intervals)
☐	☒	For null hypothesis testing, the test statistic (e.g. *F*, *t*, *r*) with confidence intervals, effect sizes, degrees of freedom and *P* value noted<br>*Give P values as exact values whenever suitable.*
☒	☐	For Bayesian analysis, information on the choice of priors and Markov chain Monte Carlo settings
☒	☐	For hierarchical and complex designs, identification of the appropriate level for tests and full reporting of outcomes
☒	☐	Estimates of effect sizes (e.g. Cohen's *d*, Pearson's *r*), indicating how they were calculated

Our web collection on statistics for biologists contains articles on many of the points above.

Software and code

Policy information about availability of computer code

Data collection	Axograph X v1.5.4 was used for the acquisition and analysis of electrophysiological recordings. Viewer v3.0.1.442 (BIOBSERVE) was used for automated behavioral analysis and ImageJ (FIJI) v2.1.0/1.53g was used for image processing and analysis.
Data analysis	Statistical analyses were performed using Prism 9.0 (GraphPad Software).

For manuscripts utilizing custom algorithms or software that are central to the research but not yet described in published literature, software must be made available to editors/reviewers. We strongly encourage code deposition in a community repository (e.g. GitHub). See the Nature Research guidelines for submitting code & software for further information.

Data

Policy information about availability of data

All manuscripts must include a data availability statement. This statement should provide the following information, where applicable:

- Accession codes, unique identifiers, or web links for publicly available datasets
- A list of figures that have associated raw data
- A description of any restrictions on data availability

The source data underlying Figs 1c-d, f-g, 2c-d, f-g, 3b-c, e-f, 4e-f, h-i, 5b-f, and 6b-d, f-h, and Supplementary Figs 1a-b, 3c-e, 5d-f, 6b-c, e-f, 7b, 7d-g, 9b-c, 10c-d, f-g, 11a-d, 12a-b, and 13b-c are provided as a Source Data file. All data are available from the corresponding author upon reasonable request.

Field-specific reporting

Please select the one below that is the best fit for your research. If you are not sure, read the appropriate sections before making your selection.

Life sciences study design

All studies must disclose on these points even when the disclosure is negative.

Sample size	No statistical methods were used to predetermine sample sizes, which were based on work in previous publications (refs. Halpern, et al., 2013; Zhu, et al., 2016). Sample size of 5–15 animals were sufficient to determine significance both in behavior tests and electrophysiological recordings.
Data exclusions	Out of the greater than 200 animals used in our experiments, <3% were excluded after euthanasia because of lack of detectable transgene expression in the targeted brain region, <1% were exclude due to aberrant behavior in CPP assays as detailed in methods, based on pre-established exclusion criteria stated in the methods.
Replication	Figure 2 was completely replicated once.
Randomization	Animals were randomized by cage prior to surgeries. For example, if there were 30 mice in an experiment, with five mice per cage, mice were randomly assigned to be in eYFP or ChR2 groups in a counterbalanced fashion.
Blinding	All experiments were conducted in a blind manner such that assays were conducted and analyzed without knowledge of the specific manipulation being performed or the genotype of the animal being studied.

Reporting for specific materials, systems and methods

We require information from authors about some types of materials, experimental systems and methods used in many studies. Here, indicate whether each material, system or method listed is relevant to your study. If you are not sure if a list item applies to your research, read the appropriate section before selecting a response.

Materials & experimental systems

n/a	Involved in the study	
☐	☒	Antibodies
☒	☐	Eukaryotic cell lines
☒	☐	Palaeontology
☐	☒	Animals and other organisms
☒	☐	Human research participants
☒	☐	Clinical data

Methods

n/a	Involved in the study	
☒	☐	ChIP-seq
☒	☐	Flow cytometry
☒	☐	MRI-based neuroimaging

Antibodies

Antibodies used

Primary: chicken anti-GFP, 1:2000, Aves labs (GFP-1020); rabbit anti-cFos 1:500 (Synaptic Systems, 226003), rat anti-mCherry 1:1500 (Invitrogen, M11217), rabbit anti-GAD65 1:1000 (ThermoFisher,PA5-22260).

Secondary: ThermoFisher Alexa Fluor: donkey anti-chicken (SA1-7200), donkey anti-rabbit 594 (A32754), donkey anti-rat 594 (A-21209).

Validation

From manufacture: 1) GFP - "Antibodies were analyzed by western blot analysis (1:5000 dilution) and immunohistochemistry (1:500 dilution) using transgenic mice expressing the GFP gene product. Western blots were performed using BlokHen® (Aves Labs) as the blocking reagent, and HRP-labeled goat anti-chicken antibodies (Aves Labs, Cat. #H-1004) as the detection reagent. Immunohistochemistry used tetramethyl rhodamine-labeled anti-chicken IgY."

3) mCherry - "mCherry Antibody (M11217) in IHC Intestinal tissue from a transgenic mouse expressing mCherry in all tissues was isolated and fixed in 4% paraformaldehyde. mCherry Rat Monoclonal Antibody (M11217) was used at a 1:15,000 dilution. Using the ImmPRESS™ Anti-Rat Ig (peroxidase) Polymer Detection Kit (Vector Laboratories) and following the manufacturer's instructions, the sections were incubated in peroxidase substrate solution until the desired stain intensity developed. A) Fluorescent image detecting mCherry expression and B) HRP-stained image."

4) GAD65 - Immunocytochemistry-Immunofluorescence analysis of GAD65 was performed in DIV9 rat E18 primary hippocampal neuron cells fixed in 4% paraformaldehyde at RT for 15 min. Green: GAD65 Polyclonal Antibody (Product # PA5-22260) diluted at 1:500. Red: beta Tubulin 3/ Tuj1, stained by beta Tubulin 3/ Tuj1 antibody. Blue: Fluoroshield with DAPI

References:

Antibodies 1-3:

Steinberg et al., Amygdala-Midbrain Connections Modulate Appetitive and Aversive Learning, Neuron 106, 1-18, 2020

1) GFP

Efrain A. Ribeiro, Alexander R. Nectow, Lisa E. Pomeranz, Mats I. Ekstrand, Ja Wook Koo, Eric J. Nestler (2019), 'Viral labeling of neurons synaptically connected to nucleus accumbens somatostatin interneurons.' Plos One. 10.1371/journal.pone.0213476.

2) cFos
The anterior insular cortex unilaterally controls feeding in response to aversive visceral stimuli in mice.
Wu Y, Chen C, Chen M, Qian K, Lv X, Wang H, Jiang L, Yu L, Zhuo M, Qiu S
Nature communications (2020) 111: 640. 226 003 IHC; tested species: mouse

3) mCherry
Heiss JE, Yamanaka A, Kilduff TS. Parallel Arousal Pathways in the Lateral Hypothalamus. eNeuro. 2018;5 (4):ENEURO.0228-18.2018. Published 2018 Aug 21. doi:10.1523/ENEURO.0228-18.2018

4) GAD65
Martínez JJ, Rahsepar B, White JA. Anatomical and Electrophysiological Clustering of Superficial Medial Entorhinal Cortex Interneurons. eNeuro. 2017;4(5):ENEURO.0263-16.2017. Published 2017 Oct 16. doi:10.1523/ENEURO.0263-16.2017

Animals and other organisms

Policy information about studies involving animals; ARRIVE guidelines recommended for reporting animal research

Laboratory animals	Male 7-16 week old C57Bl/6 mice (Jackson Laboratory), Slc17a6tm2(cre)Lowl/J (VGLUT2-Cre, Jackson Laboratory), B6.Cg-Tg (Drd1a-tdTomato)6Calak/J (D1-tdTomato, Jackson Laboratory), and B6;FVB-Tg(Drd2-EGFP/Rpl10a)CP101Htz/J (D2-eGFP, Jackson Laboratory) were housed on a 12-hour light-dark cycle with food and water ad libitum.
Wild animals	Did not involve
Field-collected samples	Did Not involve
Ethics oversight	All procedures complied with the animal care standards set forth by the National Institute of Health and were approved by Stanford University's Administrative Panel on Laboratory Animal Care and Administrative Panel of Biosafety.

Note that full information on the approval of the study protocol must also be provided in the manuscript.
